# Marrow leptin-LEPR signaling rewires mitochondrial oxidative metabolism to confer chemoresistance in acute myeloid leukemia

**DOI:** 10.1038/s41419-026-08528-0

**Published:** 2026-02-23

**Authors:** Xinai Liao, Wei Dai, Xiaolin Xu, Danni Cai, Maoqing Tan, Zukai Wang, Yanrong Huang, Diyu Hou, Jingru Liu, Liuhuan Wang, Jin Wang, Xiaoting Wang, Shuxia Zhang, Xinjian Lin, Huifang Huang

**Affiliations:** 1 Central Laboratory, Fujian Medical University Union Hospital, Fuzhou, Fujian 350001 China; 2 Department of Colorectal Surgery, the First Affiliated Hospital, Fujian Medical University, Fuzhou, Fujian 350005 China; 3 Fujian Institute of Hematology, Fujian Provincial Key Laboratory on Hematology, Fujian Medical University Union Hospital, Fuzhou, Fujian 350001 China; 4 Key Laboratory of Gastrointestinal Cancer (Fujian Medical University), Ministry of Education, Fuzhou, Fujian 350122 China

**Keywords:** Cancer therapy, Prognostic markers

## Abstract

Leptin is abundant within marrow adipose tissue, yet its impact on acute myeloid leukemia (AML) therapy response is undefined. Here, we report that elevated bone-marrow leptin and blast-cell leptin-receptor (LEPR) levels strongly associate with poor cytarabine (Ara-C) clearance and reduced survival in newly diagnosed AML patients. Mechanistic and functional validation in human AML lines, primary blasts, and two syngeneic mouse models (MLL-AF9, AML1-ETO9a) shows that exogenous leptin markedly blunts Ara-C cytotoxicity, whereas the high-affinity LEPR antagonist Allo-aca restores chemosensitivity without altering baseline leukemia growth. Leptin up-regulates LEPR and triggers JAK2/STAT3 signaling that boosts mitochondrial complex Ⅰ activity, oxidative phosphorylation, and mitochondrial reactive oxygen species (mtROS); the resulting mtROS surge activates a compensatory antioxidant program that shields blasts from drug-induced oxidative damage. These data identify an adipokine-driven metabolic circuit governing AML chemoresistance and reveal LEPR blockade as a tractable strategy to improve outcomes, underscoring adipose–tumor crosstalk as a general therapeutic vulnerability.

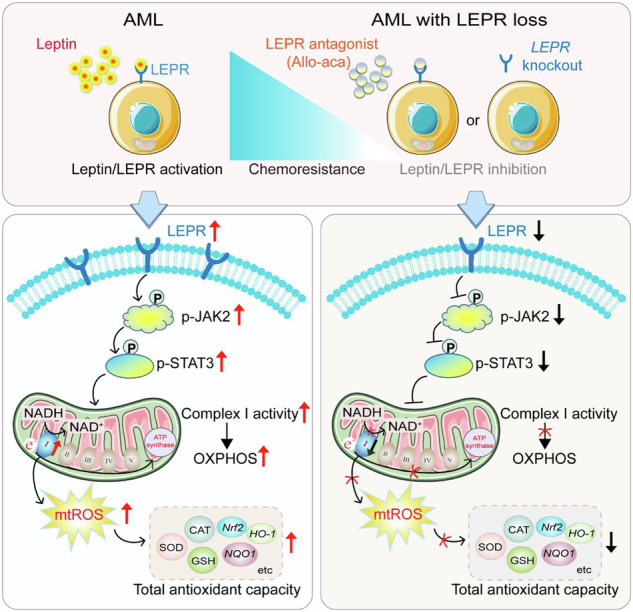

## Introduction

Acute myeloid leukemia (AML) is an aggressive hematologic malignancy characterized by the infiltration and expansion of immature myeloid blasts, disrupting normal hematopoiesis. Despite intensive chemotherapy, patient outcomes remain poor, with high relapse rates underscoring the critical challenge of therapeutic resistance [1–3].

Growing evidence indicates that the bone-marrow niche actively shapes therapeutic response, with adipocytes emerging as key regulators of leukemic survival [4]. These cells not only accumulate free fatty acids and release tumor-supportive adipokines but also transfer high-energy lipids to fuel the mitochondrial metabolism that underpins treatment resistance and disease progression [5]. Among adipocyte-derived factors, leptin—best known for controlling systemic energy balance [6–8]—has attracted attention because its dual role in modulating oncogenic processes and metabolic dysregulation. Leptin mediates its pleiotropic effects by binding to its receptor (LEPR) and couples to oncogenic JAK/STAT3, PI3K/AKT, and MAPK/ERK pathways [9, 10]. LEPR is frequently over-expressed on cancer cells suggests its potential as a biomarker [11, 12]. Critically, leptin–LEPR activation has been linked to drug resistance in several solid tumors through mechanisms including fatty acid β-oxidation (FAO), mitochondrial fusion and cancer stem cell self-renewal, yet its role in AML chemoresistance remains undefined [13–15], yet its role in AML chemoresistance remains undefined. While our prior work demonstrated that bone marrow adipocytes confer chemoresistance in AML [16], the specific contribution of leptin-LEPR signaling to this process warrants further investigation.

Reactive oxygen species (ROS) exert context-dependent effects in cancer: modest increases can stimulate proliferation and genomic instability that favor tumorigenesis, whereas excessive ROS trigger oxidative damage and cell death [17]. Many chemotherapeutics, including cytarabine (Ara-C), exploit this vulnerability by further elevating intracellular ROS. Tumor cells, however, counteract oxidative stress by up-regulating antioxidant networks such as glutathione synthesis and the NFE2-related factor 2 (Nrf2)/ heme oxygenase-1 (HO-1) axis to restore redox homeostasis and sustain viability [18, 19]. Heightened antioxidant capacity is now recognized as a common mechanism of chemotherapy resistance in multiple malignancies, including AML[20–25]. For example, the *FLT3-ITD* oncogene drives ROS production that secondarily activates Nrf2/HO-1, thereby promoting drug resistance in AML [24], while Nestin^⁺^ bone-marrow mesenchymal stem cells enhance glutathione recycling to protect leukemic blasts from cytotoxic injury [25]. These observations highlight antioxidant rewiring as an actionable therapeutic target. AML cells depend heavily on mitochondrial oxidative phosphorylation (OXPHOS)—the principal intracellular ROS source—to satisfy their metabolic demands [26]. Yet how leukemic blasts buffer the additional ROS burden imposed by OXPHOS remains poorly defined.

Here, we report that newly diagnosed AML patients with high bone-marrow leptin and elevated blast-cell LEPR exhibit poor Ara-C clearance and reduced survival. In two syngeneic mouse models and human AML cell lines, leptin up-regulates LEPR and activates JAK2/STAT3, boosting mitochondrial complex Ⅰ activity, OXPHOS, and mitochondrial ROS (mtROS). The ensuing mtROS surge induces a compensatory antioxidant response that shields blasts from cytotoxic stress, whereas pharmacologic (Allo-aca) or genetic *LEPR* blockade collapses this defense and restores chemosensitivity. Our findings uncover an adipokine-driven metabolic circuit that underlies AML drug resistance and establish LEPR antagonism as a tractable strategy to improve patient outcomes.

## Materials and methods

### Primary AML samples

A total of 84 bone marrow aspirates were collected from newly diagnosed AML patients (excluding APL) with informed consent at Fujian Medical University Union Hospital. This study was conducted in accordance with the Declaration of Helsinki guidelines and approved by the Ethics Committee of Fujian Medical University Union Hospital ([2024] XY-229). Bone marrow mononuclear cells (BMMNCs) were isolated by density gradient centrifugation with Lymphocyte Separation Medium (TBD science, Tianjin, China). Clinical characteristics of patients are provided in Table S1.

### Animal experiments

Male C57BL/6 J (CD45.2) mice (8-10 weeks old) were purchased from GemPharmatech (Nanjing, Jiangsu, China) and maintained under specific pathogen-free (SPF) barrier conditions. We did not employ statistical methods for sample size estimation or blinding in the experiments. Animal exclusion criteria were not defined, and randomization was used for group allocation. All experimental protocols were conducted in accordance with the National Institutes of Health Guide for the Care and Use of Laboratory Animals and were approved by the Institutional Animal Care and Use Committee of Fujian Medical University (IACUCFJMU 2024-Y-0088).

MLL-AF9 AML cells were generated as previously described [16]. Briefly, the retroviral constructs MSCV-MLL-AF9-IRES-YFP (30 μg) along with the packaging plasmid pCL-ECO (15 μg) were transfected into HEK293T cells using Lipofectamine 3000 (Invitrogen, Carlsbad, CA, USA) for viral packaging. Lineage-negative fetal liver cells isolated from C57BL/6 J embryos were transduced with the MLL-AF9 retrovirus and then intravenously injected into sublethal irradiation (6.5 Gy) C57BL/6 J mice. YFP^+^ bone marrow cells were sorted from primary recipient mice by fluorescence-activated cell sorting (FACS) and transplanted into non-irradiated secondary recipients at a dose of 2 × 10⁵ cells per mouse. GFP-labeled AML1-ETO9a leukemia cells were kindly provided by Prof. Jianxiang Wang (Institute of Hematology and Hospital of Blood Diseases, Chinese Academy of Medical Sciences & Peking Union Medical College). To establish AML1-ETO9a AML mouse model, 8 × 10^5^ GFP^+^ leukemia cells were transplanted into the irradiated (4.5 Gy) recipient mice.

To assess the impact of leptin and Allo-aca on chemotherapy sensitivity in AML, AML mice were randomly assigned to several groups and subjected to the following therapeutic regimens: (i) MLL-AF9-induced leukemic mice received daily intraperitoneal injections of leptin (300 ng/kg; R&D Systems, Minneapolis, MN, USA) or Allo-aca (1 mg/kg; MedChemExpress, Monmouth Junction, NJ, USA) from days 11 to 20. Subsequently, Ara-C (100 mg/kg; Cytosar, Foshan, Guangdong, China) was co-administered intraperitoneally from days 16 to 20. (ii) In the AML1-ETO9a leukemic model, mice were treated with leptin or Allo-aca (same dosage as above) from days 14 to 23, with concurrent Ara-C administration on days 19 to 23 to assess combination therapy efficacy.

### Correlation between marrow leptin levels and chemotherapy response

Chemotherapy outcomes was assessed using two primary indicators: bone marrow blasts clearance rate and chemotherapy efficacy, categorized as objective response rate (ORR) and non-response (NR). The blast clearance was calculated as the percentage reduction in blast ratio from baseline diagnosis (P1) to 4 weeks post-treatment (P2) using the formula: Clearance rate (%) = [(P1 - P2) / P1] × 100%. Chemotherapy efficacy was evaluated during hematopoietic recovery (~4 weeks post-induction) based on: (i) bone marrow blast percentage, (ii) hematologic recovery (absolute neutrophil count [ANC] and platelet count) and (iii) extramedullary disease status [27], with ORR comprising complete remission (CR; <5% blasts, no extramedullary disease, platelets ≥100×10⁹/L, and ANC ≥ 1.0×10⁹/L), CR with incomplete recovery (CRi; meeting CR criteria except for platelets <100×10⁹/L or ANC < 1.0×10⁹/L), or partial remission (PR; 5-25% blasts with ≥50% reduction from baseline and platelets/ANC meeting CR thresholds), while NR represented failure to achieve these responses. Marrow plasma leptin concentrations were measured using a Human Leptin ELISA Kit (Cusabio, Wuhan, Hubei, China), with Spearman’s correlation (*r*) analyzing leptin-blast clearance relationships and comparative analyses examining leptin levels between ORR and NR groups. Predictive performance was assessed through receiver operating characteristic (ROC) curve analysis, with area under the curve (AUC) values reported alongside 95% confidence intervals. Logistic regression analyzed leptin concentration, age, peripheral white blood cell (WBC) count, marrow blast percentage and ELN 2022 risk classification impacts on efficacy, with results expressed as odds ratios (OR) and *p*-values.

### CRISPR/cas9-mediated *LEPR* knockout cell lines

*LEPR* knockout cells were generated by the CRPSR/cas9 system. Specific single-guide RNA (sgRNA) sequences (Table S2) targeting the *LEPR* were designed and cloned into the GV708 (U6-sgRNA-EF1a-Cas9-FLAG-CMV-EGFP-P2A-puro) vector (Genechem, Shanghai, China). Recombinant lentiviruses were produced in HEK293T cells by co-transfecting the sgRNA plasmid with psPAX2 and pMD2.G packaging plasmids at a 4:3:1 ratio using Lipofectamine 3000. Virus-containing supernatants were collected at 48 h and 72 h post-transfection, centrifuged at 2,000 rpm for 10 min at 4°C, and filtered through a 0.45 μM low protein binding membrane (Merck Millipore, Burlington, MA, USA). The lentiviruses transduced AML cell lines (U937, HL-60, and THP-1) in the presence of polybrene (10 µg/mL; Dingguo, Beijing, China) for 72 h. Following puromycin (1 µg/mL; Solarbio, Beijing, China) selection, *LEPR* knockout efficiency was confirmed by western blot analysis.

### Statistical analysis

All statistical analyses were conducted using R software (version 4.1.3; R Foundation for Statistical Computing, Vienna, Austria) and GraphPad Prism 9 (GraphPad Software Inc, San Diego, CA, USA). Data distribution was evaluated using the Kolmogorov-Smirnov normality test. The groups being statistically compared showed homogeneity of variance. Between-group differences were analyzed using two-tailed unpaired t tests, paired t test or chi-squared tests. One-way ANOVA was used for multiple group comparisons. Statistical significance for survival was calculated by the log-rank test. *p* < 0.05 were considered statistically significant.

Additional materials and methods are included in the Supplementary materials.

## Results

### High marrow leptin and blast-cell LEPR identify AML patients with poor response and survival

**Fig. 1:**
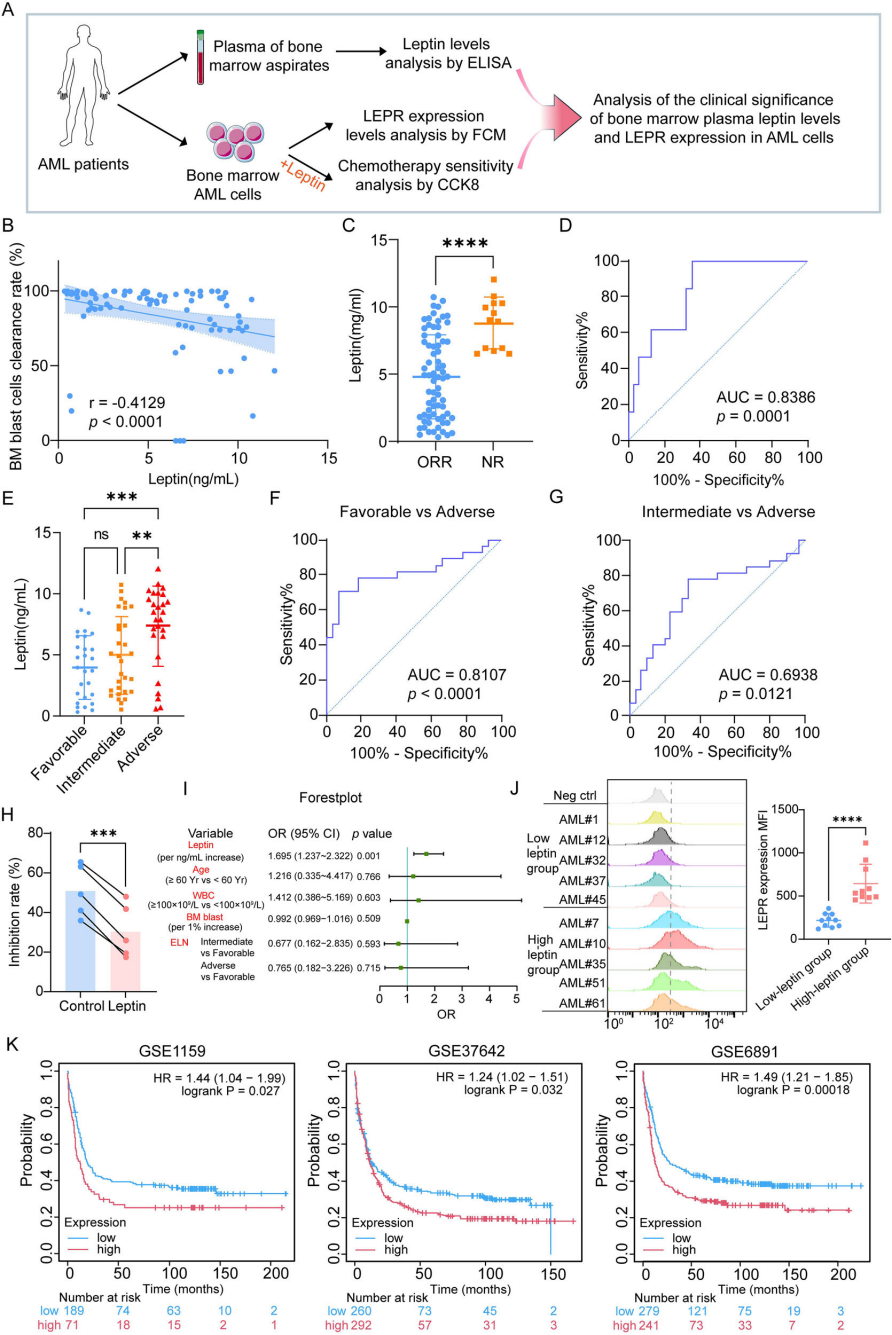
High marrow leptin and blast-cell LEPR identify AML patients with poor response and survival. **A** Schematic diagram of clinical significance analysis for bone marrow (BM) plasma leptin levels and blast-cell LEPR expression in 84 newly diagnosed AML patients. **B** BM plasma leptin levels correlate inversely with blast clearance rates in AML patients. **C** Leptin concentrations in BM plasma from AML patients with differential chemotherapeutic responses. **D** ROC curve of leptin in BM plasma for distinguishing ORR and NR groups. **E** Leptin levels in the BM plasma of AML patients stratified by ELN 2022 risk classification. **F** ROC curve of marrow plasma leptin for distinguishing between favorable and adverse risk AML groups. **G** ROC curve of marrow plasma leptin for distinguishing between intermediate and adverse risk AML groups. **H** CCK-8 analysis of cell viability in leptin-pretreated primary AML cells following 24 h Ara-C exposure (*n* = 5). **I** The forest plot shows the results of logistic regression analysis. **J** Representative FCM profiles and MFI statistical analysis of LEPR expression in BM leukemia cells from high- and low-leptin groups (*n* = 10 patients per group). MFI: mean fluorescence intensity. **K** Kaplan-Meier analysis of overall survival in AML patients stratified by LEPR expression levels from GSE1159, GSE37642 and GSE6891 datasets. Data are presented as mean ± SD **C, E, J**. Significance differences were determined by two-tailed unpaired t test with Welch’s correction **C**, one-way ANOVA with Holm-Šídák’s multiple comparisons test (E), two-tailed paired t test **H**, or two-tailed unpaired t test **J**. ns, not significant, **p < 0.01, ***p < 0.001, ****p < 0.0001.

To determine the clinical relevance of marrow microenvironment-derived leptin in AML, we prospectively measured bone-marrow plasma leptin in 84 newly diagnosed adults (Fig. 1A) and dichotomized the cohort at the median value (5.471 ng/mL). Baseline sex, age, blood counts, blast percentage, FAB class, and mutational profile were balanced between high- and low-leptin groups, yet ELN-2022 risk stratification [27] was skewed: adverse-risk disease was three-times more common in the high-leptin arm (50.0% vs. 14.3%), whereas favorable-risk cases predominated in the low-leptin arm (42.9% vs. 21.4%; Table 1), suggesting leptin’s association with aggressive AML phenotypes.

**Table 1:** Clinical characteristics of AML patients in the high and low leptin groups.

Patient’s parameters	Low (leptin < 5.47 ng/mL, *n* = 42)	High (leptin ≥ 5.47 ng/mL, *n* = 42)	*p* value
Sex, male/female	21/21	25/17	0.381
Age, years (X ± SD)	48.64 ± 18.49	47.76 ± 15.38	0.813
Median WBC, ×10^9^/L (range)	34.37 (0.75-277.14)	23.77 (1.03-291.44)	0.727
Median PB blasts, % (range)	48 (0-98)	27 (0-95)	0.110
Median BM blasts, % (range)	43 (3-96)	39.5 (4-91)	0.250
FAB			0.643
M0	0	1	1.000
M1	6	3	0.483
M2	13	13	1.000
M4	1	0	1.000
M5	22	25	0.510
Karyotypes			0.307
normal	30	22	0.072
t (8;21)	8	7	0.776
t (9;22)	0	2	0.494
+8	1	4	0.360
11q23	0	3	0.241
inv (16)	1	1	1.000
inv (3)	0	1	1.000
others	0	1	1.000
complex	2	1	1.000
Risks (ELN 2022)			0.002
Good	18	9	0.035
Intermediate	18	12	0.172
Poor	6	21	<0.001
Gene fusion (+/-)	11/31	18/24	0.108
Gene mutations (+/-)	35/7	39/3	0.178
*FLT3* (+/-)	12/30	10/32	0.620
*NPM1* (+/-)	10/32	7/35	0.415
*DNMT3A* (+/-)	6/36	4/38	0.500
*IDH2* (+/-)	7/35	3/39	0.178
*IDH1* (+/-)	1/41	3/39	0.616
*TET2* (+/-)	1/41	7/35	0.057
*RUNX1* (+/-)	3/39	4/38	1.000
*NRAS* (+/-)	2/40	5/37	0.433
*CEBPA* (+/-)	7/35	9/33	0.578
*WT1* (+/-)	2/40	3/39	1.000
*KIT* (+/-)	7/35	4/38	0.332
*GATA2* (+/-)	2/42	5/37	0.433
*STAG2* (+/-)	2/40	3/39	1.000
*ROBO2* (+/-)	2/40	1/41	1.000
*RELN* (+/-)	2/40	1/41	1.000
*ARID2* (+/-)	2/40	1/41	1.000
*ASXL1* (+/-)	3/39	6/36	0.483

*WBC* white blood cell, *PB* peripheral blood, *BM* bone marrow, *FAB* French-American-British classification, *ELN 2022* European LeukemiaNet 2022 risk classification.

Spearman correlation analysis revealed that marrow leptin inversely correlated with day-14 blast clearance (Spearman *r* = –0.41, *p* < 0.0001; Fig. 1B). Non-responders exhibited a twofold higher median leptin than responders (9.03 vs. 4.81 ng/mL; Fig. 1C), and leptin accurately predicted induction failure (AUC = 0.84; Fig. 1D). Concentrations were highest in ELN adverse-risk AML (median 8.53 ng/mL) and distinguished adverse from favorable disease with an AUC of 0.81 (Fig. 1E–G). In primary blasts leptin itself did not alter viability (Fig. S1), yet it attenuated Ara-C (a commonly used chemotherapeutic agent in AML treatment) cytotoxicity (Fig. 1H). Logistic regression confirmed leptin as an independent predictor of chemoresistance (OR = 1.70, 95% CI 1.24–2.32; Fig. 1I). Because leptin signals via its receptor, we quantified LEPR on CD34⁺ CD45⁺ blasts by flow cytometry (FCM). Surface LEPR was significantly higher in high-leptin patients (Fig. 1J). Pooled transcriptomic data from three external AML cohorts (GSE1159, GSE37642, GSE6891) showed that high LEPR expression portends inferior overall survival (Fig. 1K). Together, these findings establish marrow leptin and blast-cell LEPR as mechanistically linked biomarkers that forecast poor therapeutic response and adverse prognosis in AML.

### Leptin–LEPR signaling limits cytarabine efficacy in AML mouse models but does not alter basal leukemia growth

Building upon our clinical findings, we further investigated whether leptin influences chemotherapeutic response and leukemic progression in vivo by transplanting syngeneic mice with AML1-ETO9a (AE9a; t(8;21)) or MLL-AF9 (MA9; t(9;11)) blasts and treating them with Ara-C alone, Ara-C plus recombinant leptin, or Ara-C plus Allo-aca—a nontoxic nine-residue peptide that antagonizes LEPR by occupying site Ⅲ [28]. Given leptin is well-established as a critical regulator of feeding behavior and energy expenditure [29], the doses chosen for our study raised bone-marrow leptin concentrations yet left body weight unchanged (Fig. S2), thereby excluding potential confounding metabolic effects.

**Fig. 2:**
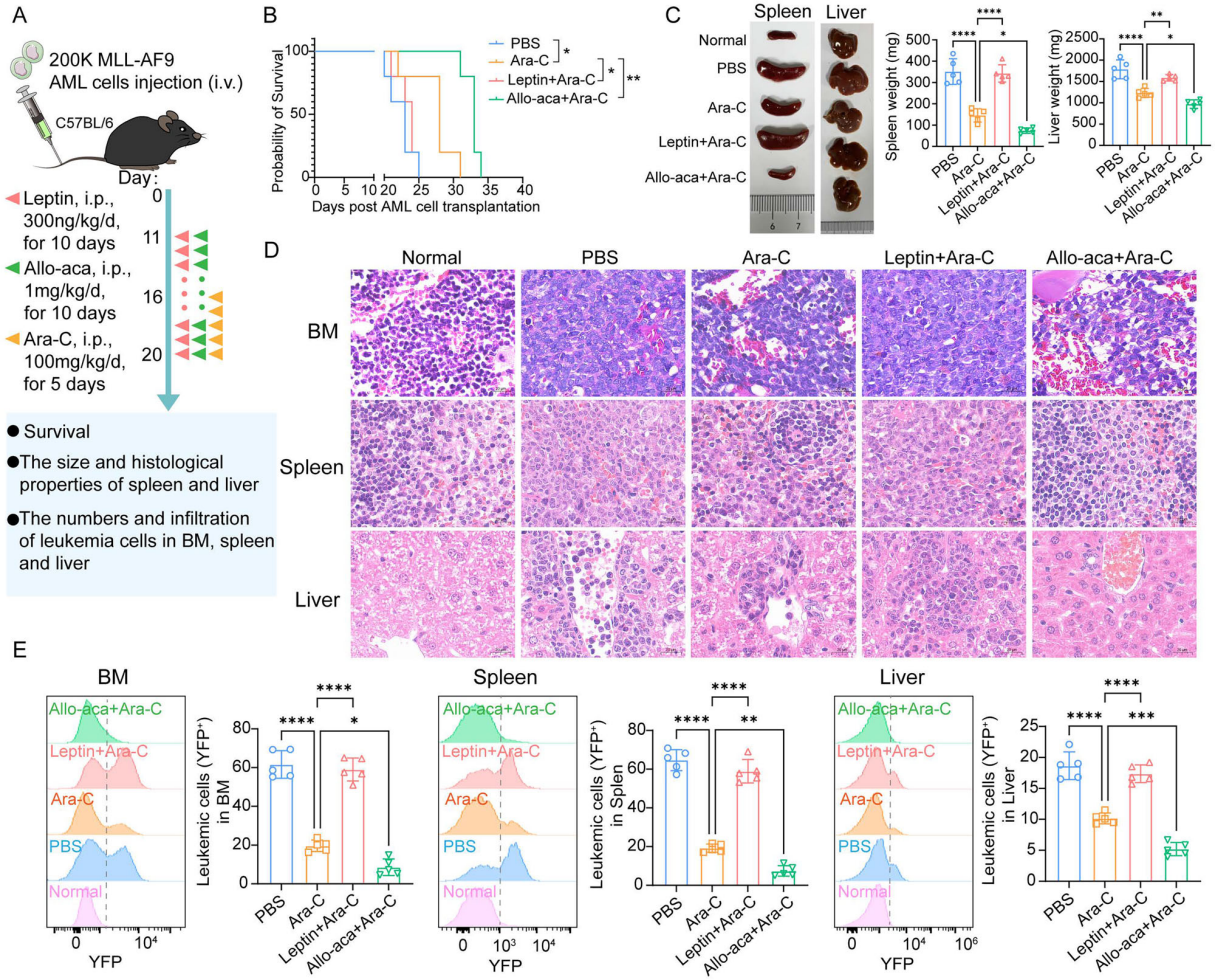
Leptin–LEPR signaling limits cytarabine efficacy in AML mouse models (MLL-AF9). **A** Experimental design of evaluating the role of leptin on the therapeutic effect in MLL-AF9-driven AML mice. **B** Kaplan-Meier survival curve of MLL-AF9 AML mice (*n* = 5 mice per group). **C** Representative images and weight comparisons of spleen and liver in MLL-AF9 AML mice treated with PBS, Ara-C, leptin + Ara-C, or Allo-aca+ Ara-C (*n* = 5 mice per group). **D** Representative H&E-stained sections of BM, spleen and liver (scale bars: 20 μm). **E** Representative FCM profiles (left) and quantification of YFP^+^ AML cells in BM, spleen, and liver of MLL-AF9 AML mice (*n* = 5 mice per group). Data are presented as mean ± SD **C, E**. Significance differences were determined by log-rank test **B**, or one-way ANOVA with Dunnett’s multiple comparisons test **C, E**. *p < 0.05, **p < 0.01, ***p < 0.001, ****p < 0.0001.

We first evaluated the impact of leptin on chemotherapeutic response in mouse models (Fig. 2A; Fig. S3A). Surprisingly, leptin co-administration significantly shortened survival relative to Ara-C monotherapy in both models, whereas Allo-aca extended survival (Fig. 2B; Fig. S3B). Concurrently, leptin dampened Ara-C cytotoxicity, producing pronounced hepatosplenomegaly and extensive leukemic infiltration of liver and spleen (Fig. 2C, D; Fig. S3C, D). Conversely, LEPR blockade with Allo-aca reversed these changes, reducing organ enlargement and limiting tissue infiltration. Moreover, FCM confirmed higher leukemic burden in marrow, spleen, and liver with leptin and reduced burden with Allo-aca (Fig. 2E; Fig. S3E). We subsequently investigated whether leptin modulates AML progression (Fig. S4A, E). Importantly, in the absence of chemotherapy, neither leptin nor Allo-aca affected leukemic development. This was evidenced by similar survival (Fig. S4B, F), organ size (Fig. S4C, G), or tissue blast counts (Fig. S4D, H). These findings indicate that leptin-LEPR signaling specifically governs chemosensitivity rather than basal proliferation in vivo.

### Leptin augments antioxidant defenses that underlie chemoresistance

**Fig. 3:**
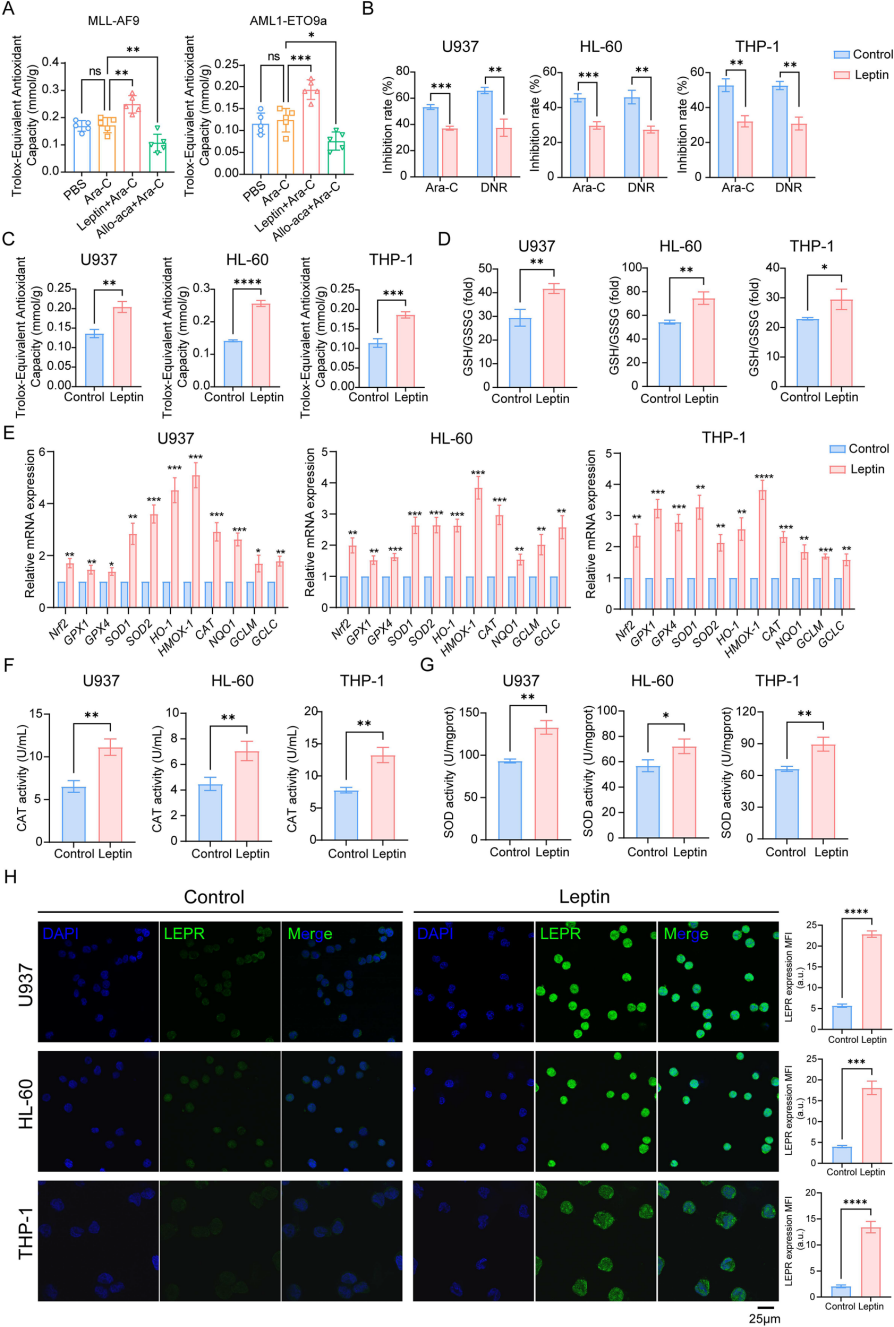
Leptin augments antioxidant defenses that underlie chemoresistance. **A** Total antioxidant capacity analysis of blast-cell from MLL-AF9 (left) and AML1-ETO9a (right) AML mice (*n* = 5 mice per group). **B** Effect of leptin on chemosensitivity to Ara-C and DNR in human AML cell lines (U937, HL-60, and THP-1). The inhibition rates were calculated from four treatment arms: (i) PBS control; (ii) Leptin alone; (iii) Ara-C alone (or DNR) and (iv) Leptin + Ara-C (or DNR). Data represent three independent biological experiments. **C** Analysis of total antioxidant capacity in leptin-treated AML cells. **D** GSH/GSSG ratio was examined with a GSH and GSSG assay kit. **E** RT-qPCR analysis of antioxidant-associated genes mRNA levels in leptin-treated AML cells. Analysis of CAT **F** and SOD **G** activity. **H** Representative immunofluorescence images (left) and MFI quantification (right) of LEPR expression (LEPR, green) in leptin-treated AML cells (scale bars: 25 μm). Data are presented as mean ± SD. Significance differences were determined by one-way ANOVA with Dunnett’s multiple comparisons test **A**, or two-tailed unpaired t test **B–H**. ns, not significant, *p < 0.05, **p < 0.01, ***p < 0.001, ****p < 0.0001.

Enhanced antioxidant buffering is a recognized driver of Ara-C resistance in AML [25]. To determine whether leptin mediates chemoresistance exploits this mechanism, we purified blasts from MA9 and AE9a leukemic mice after treatment. Relative to Ara-C alone, leptin + Ara-C increased total antioxidant capacity, whereas LEPR blockade with Allo-aca produced the opposite effect (Fig. 3A). We then explored whether the effect observed in primary AML mouse models were consistent in human AML cell lines under in vitro conditions. As expected, human AML cell lines recapitulated these findings: leptin reduced Ara-C or daunorubicin (DNR) cytotoxicity in CCK-8 assays (Fig. 3B), and concomitantly raised both total antioxidant capacity (Fig. 3C) and the reduced glutathione/oxidized glutathione (GSH/GSSG) ratio (Fig. 3D). Furthermore, at the transcript level, leptin up-regulated a broad panel of redox genes, including *Nrf2*, glutathione peroxidase 1 (*GPX1*), glutathione peroxidase 4 (*GPX4*), superoxide dismutase 1 (*SOD1*), superoxide dismutase 2 (*SOD2*), *HO-1*, heme oxygenase 1 (*HMOX-1*), catalase (*CAT*), NAD(P)H: quinone oxidoreductase 1 (*NQO1*), glutamate-cysteine ligase, catalytic subunit (*GCLC*) and glutamate-cysteine ligase modifier subunit (*GCLM*) (Fig. 3E). Additionally, enzymatic activities of CAT and SOD were likewise elevated in leptin-treated AML cells (Fig. 3F, G). Notably, immunofluorescence revealed that leptin also increased LEPR abundance in AML cells (Fig. 3H), suggesting a positive feedback loop that could amplify downstream signaling. Together, these data show that leptin reinforces antioxidant program, thereby diminishing Ara-C efficacy in AML.

### Upregulation of LEPR is key to leptin-mediated chemoresistance

**Fig. 4:**
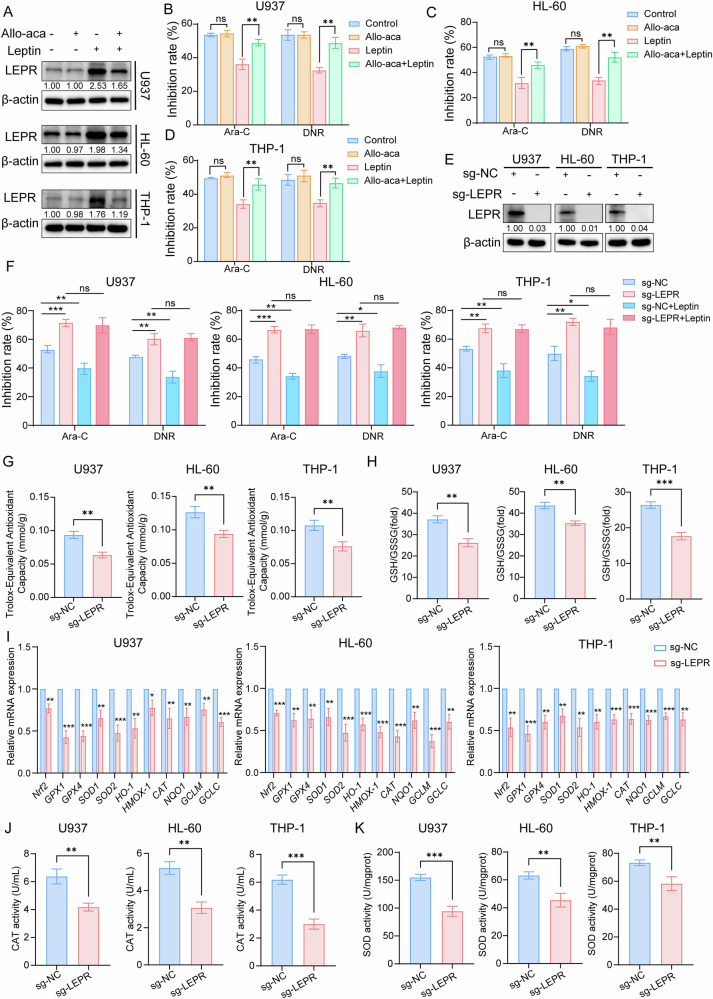
Upregulation of LEPR is key to leptin-mediated chemoresistance. **A** Western blot analysis of LEPR expression in AML cells under indicated treatments. β-actin served as the loading control. Band intensities were quantified using ImageJ software and normalized to the control group. **B–D** Effect of Allo-aca on AML cell chemosensitivity by CCK-8 assay. The inhibition rates were calculated from eight treatment arms: (i) PBS; (ii) Allo-aca alone; (iii) Leptin alone; (iv) Allo-aca + Leptin; (v) Ara-C alone (or DNR); (vi) Allo-aca + Ara-C (or DNR); (vii) Leptin + Ara-C (or DNR) and (viii) Allo-aca + Leptin + Ara-C (or DNR). Data represent three independent biological replicates. **E** Western blot to confirm the depletion of *LEPR* in AML cells. β-actin served as a loading control. **F** Chemosensitivity analysis of *LEPR*-knockout AML cells with or without leptin treatment. Comparison of total antioxidant capacity **G**, GSH/GSSG ratio **H**, antioxidant-related genes mRNA levels **I**, CAT **J** and SOD **K** activity in AML cells with or without *LEPR* knockout. Data are presented as mean ± SD **B–D, F–K**. Significance differences were determined by two-tailed unpaired t test. ns, not significant, *p < 0.05, **p < 0.01, ***p < 0.001.

Immunoblotting analysis confirmed that leptin elevates LEPR expression in AML cells, whereas the antagonist Allo-aca, although inert on its own, fully prevented this up-regulation (Fig. 4A). Concordantly, Allo-aca alone did not alter Ara-C sensitivity but consistently reversed leptin-mediated resistance in multiple AML cell lines (Fig. 4B–D). To explore the functional contribution of LEPR abundance to drug response, we ablated *LEPR* with CRISPR/Cas9 (Fig. 4E). Surprisingly, LEPR-null cells displayed markedly greater sensitivity to chemotherapy than control cells in the absence of leptin, and addition of leptin failed to rescue this effect (Fig. 4F), indicating that the receptor itself, not downstream ligand engagement, is the dominant determinant of chemosensitivity. To mechanistically interrogate this finding, we performed systematic characterization of the antioxidant defense network in *LEPR*-deficient AML cells. Subsequent experiments revealed that loss of LEPR impaired the redox buffering program that underlies resistance. Compared with wild-type cells, *LEPR*-knockout AML cells showed lower total antioxidant capacity (Fig. 4G), a reduced GSH/GSSG ratio (Fig. 4H), broad down-regulation of antioxidant genes (Fig. 4I), and diminished CAT and SOD activities (Fig. 4J, K). These data demonstrate that cell-intrinsic LEPR expression orchestrates antioxidant defenses and is indispensable for leptin-driven chemoresistance in AML.

### Leptin boosts mitochondrial OXPHOS and drives mtROS accumulation in AML cells

**Fig. 5:**
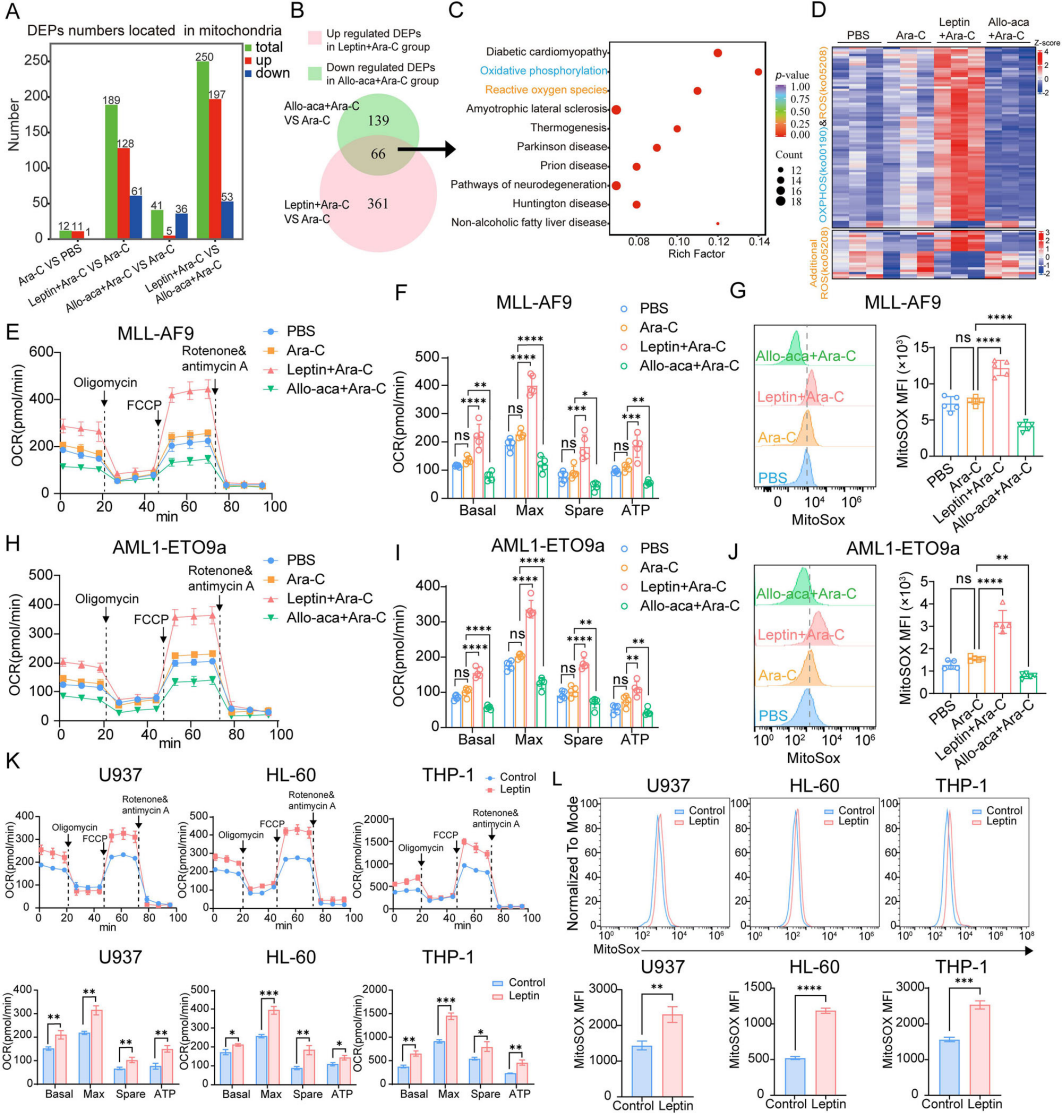
Leptin boosts mitochondrial OXPHOS and drives mtROS accumulation in AML cells. **A** Numbers of DEPs located in mitochondria of MLL-AF9 leukemia cells (*n* = 3 mice per group). **B** Venn diagram showing the overlapping DEPs that up-regulated in the leptin + Ara-C group but down-regulated in the Allo-aca + Ara-C group (versus Ara-C alone, *n* = 3 mice per group). **C** KEGG pathway enrichment analysis of **B**. **D** Heatmap analysis of OXPHOS (Ko00190) and ROS (Ko05208) pathway protein expression in MLL-AF9 leukemia cells. **E** Mitochondrial respiration analysis of sorted MLL-AF9 AML cells using Seahorse XF technology (*n* = 5 mice per group). **F** Calculation of basal respiration (Basal), maximal respiratory capacity (Max), spare respiratory capacity (Spare), and mitochondrial ATP production (ATP) in MLL-AF9 leukemia cells based on the measurements in **E**. **G** Representative FCM profiles (left) and MFI quantification (right) of mtROS levels in MLL-AF9 leukemia cells by MitoSOX staining (*n* = 5 mice per group). **H** Measurement of OCR levels in AML1-ETO9a leukemia cells. **I** Calculation of Basal, Max, Spare, and ATP in AML1-ETO9a leukemia cells based on the measurements in **H**. **J** Representative FCM profiles (left) and MFI statistical analysis (right) of mtROS levels in AML1-ETO9a leukemia cells. **K** OCR was measured (upper) and calculation of basal, Max, Spare, and ATP (lower) in leptin-treated AML cells. **L** Representative FCM histogram (upper) and MFI statistical analysis (lower) of mtROS levels in leptin-treated AML cells. Data are presented as mean ± SD **F, G, I–L**. Significance differences were determined by one-way ANOVA with Dunnett’s multiple comparisons test **F, G, I,** **J**, or two-tailed unpaired t test **K, L**. ns, not significant, *p < 0.05, **p < 0.01, ***p < 0.001, ****p < 0.0001.

To define how leptin amplifies antioxidant defenses, we performed label-free quantitative proteomics on fluorescence-activated cell sorting (FACS)-purified MA9 blasts from mice treated with Ara-C alone, leptin + Ara-C, or Allo-aca + Ara-C. Given the intimate link between mitochondrial metabolism and cellular redox homeostasis, we analyzed subcellular localization of differentially expressed proteins (DEPs). Leptin co-treatment markedly increased the number of differentially expressed mitochondrial proteins, predominantly characterized by upregulation, whereas Allo-aca produced the opposite effect (Fig. 5A).

Next, we identified sixty-six overlapping DEPs that were up-regulated by leptin but down-regulated by Allo-aca relative to Ara-C alone (Fig. 5B). KEGG pathway analysis revealed that these DEPs strong enrichment in OXPHOS and ROS-related pathways (Fig. 5C). Heatmap visualization of OXPHOS- and ROS-pathway proteins revealed that most exhibited coordinated upregulation in leptin + Ara-C–treated blasts, whereas Allo-aca co-treatment reversed these expression patterns (Fig. 5D). Gene set enrichment analysis (GSEA) further confirmed that leptin significantly activate OXPHOS and ROS pathways compared to Ara-C monotherapy, whereas Allo-aca abrogated these metabolic effects (Fig. S5). Functional assays corroborated these signatures. Seahorse metabolic analyses of marrow-sorted blasts demonstrated that leptin maintained elevated levels of OXPHOS, with significant increases in basal and maximal respiration, spare respiratory capacity, and ATP-linked O₂ consumption under Ara-C, whereas Allo-aca suppressed mitochondrial respiration and shifted cells into a hypometabolic state (Fig. 5E, F, H, I). Consistent with these findings, FCM confirmed that leptin elevated mtROS—an obligate by-product of enhanced OXPHOS—and that this increase was abolished by Allo-aca (Fig. 5G, J).

In vitro studies further validated this energy-regulatory mechanism in human AML cell lines. Leptin augmented respiration and mtROS, whereas CRISPR deletion of *LEPR* suppressed both (Fig. 5K, L; Fig. S6). Interestingly, leptin also promoted mitochondrial biogenesis, as evidenced by greater mitochondrial mass (Fig. S7A–C), increased membrane potential (ΔΨm) (Fig. S7D–F), higher mtDNA copy number (Fig. S7G), and a shift from punctate to elongated mitochondrial networks visualized by TOMM20 staining (Fig. S7H–J). Taken together, these findings indicate that leptin-LEPR signaling could potentiate antioxidant defenses in AML, possibly through metabolic reprogramming involving enhanced OXPHOS function and augmented mtROS generation.

### Leptin amplifies an mtROS-driven antioxidant circuit that fuels chemoresistance

**Fig. 6:**
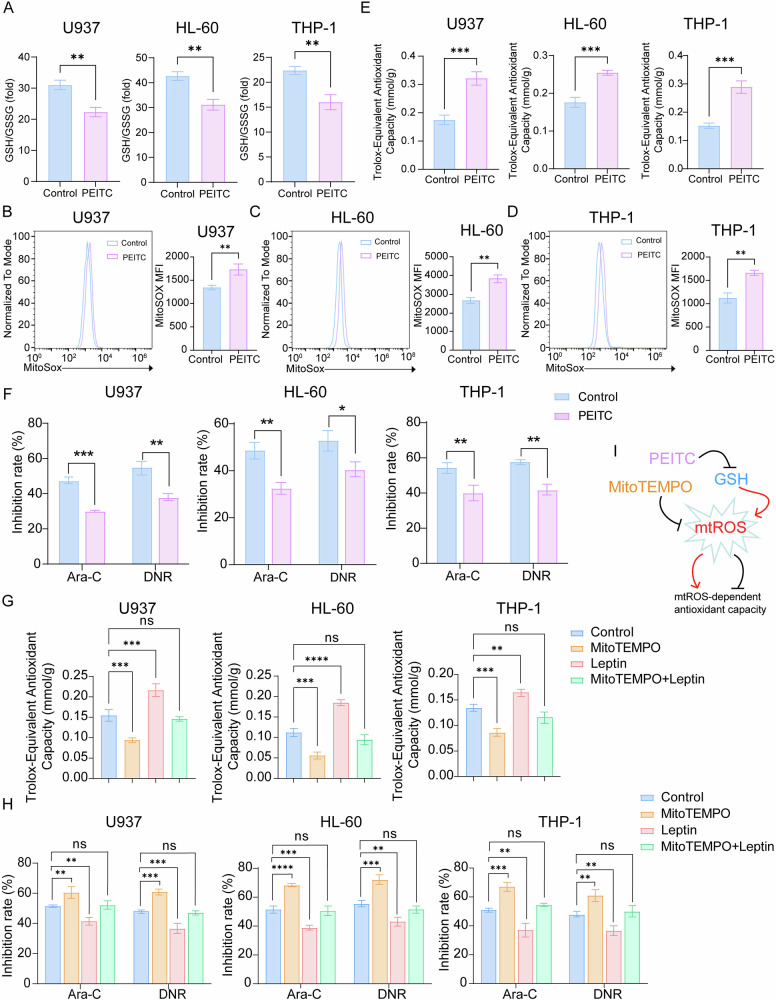
Leptin amplifies an mtROS-driven antioxidant circuit that fuels chemoresistance. **A** Analysis of GSH/GSSG ratio in PEITC-treated AML cell lines. Effect of PEITC on mtROS generation in U937 cells **B**, HL-60 cells **C** and THP-1 cells **D**. **E** Comparison of total antioxidant capacity of AML cell lines treated with or without PEITC. **F** Effect of PEITC on AML cell chemosensitivity. **G** Total antioxidant capacity in MitoTEMPO-treated AML cells. **H** CCK-8 analysis of MitoTEMPO’s effect on AML cell chemosensitivity. **I** The possible mechanisms through which PEITC or MitoTEMPO regulates mtROS-dependent antioxidant capacity in AML cells. Data are presented as mean ± SD **A–H**. Significance differences were determined by two-tailed unpaired t test **A–F**, or one-way ANOVA with Dunnett’s multiple comparisons test **G, H**. ns, not significant, *p < 0.05, **p < 0.01, ***p < 0.001, ****p < 0.0001.

The survival advantage conferred by elevated OXPHOS in AML necessitates adaptive antioxidant buffering to mitigate the resulting mtROS overload, which may reveal the more profound biological mechanism underlying this adaptation. To interrogate this dependency, we exposed AML cells to β-phenethyl isothiocyanate (PEITC), a phytochemical known to exert antitumor effects by depleting GSH and provoking oxidative stress through ROS accumulation [30]. Consistent with previous reports, PEITC treatment significantly lowered the GSH/GSSG ratio (Fig. 6A) and increased mtROS (Fig. 6B–D). Paradoxically, despite its pro-oxidant effects, PEITC simultaneously enhanced total antioxidant capacity (Fig. 6E) and notably, rendered AML cells resistant to Ara-C and DNR (Fig. 6F), implying that an acute mtROS surge can trigger compensatory antioxidant programs, ultimately confer chemoresistance. When mtROS was quenched with the mitochondria-targeted scavenger MitoTEMPO, global antioxidant defenses collapsed and chemosensitivity was restored; strikingly, leptin rescued both antioxidant capacity and drug resistance under these conditions (Fig. 6G, H). These observations place the mtROS–antioxidant axis at the center of leptin-mediated chemoprotection and identify it as a tractable therapeutic vulnerability in AML (Fig. 6I).

### JAK2/STAT3–dependent activation of complex I links leptin to mtROS-driven antioxidant defense

Given the established leptin-LEPR function through JAK2/STAT3 signaling [31, 32], we asked whether this pathway underlies leptin-mediated chemoresistance in AML. Immunofluorescence staining of marrow blasts from both MA9 and AE9a murine models revealed that leptin given with Ara-C increased LEPR abundance and phosphorylation of JAK2 and STAT3 (Y705 and S727) compared with Ara-C alone - effects that were completely abolished by Allo-aca co-treatment (Fig. S8A–F). In line with these observations, human AML cell lines exhibited leptin-mediated activation of LEPR signaling, and CRISPR/Cas9-mediated loss of *LEPR* eliminated leptin-induced phosphorylation at LEPR Y986/Y1411, JAK2, and both STAT3 sites (Y705/S727) (Fig. S8G, H).

**Fig. 7:**
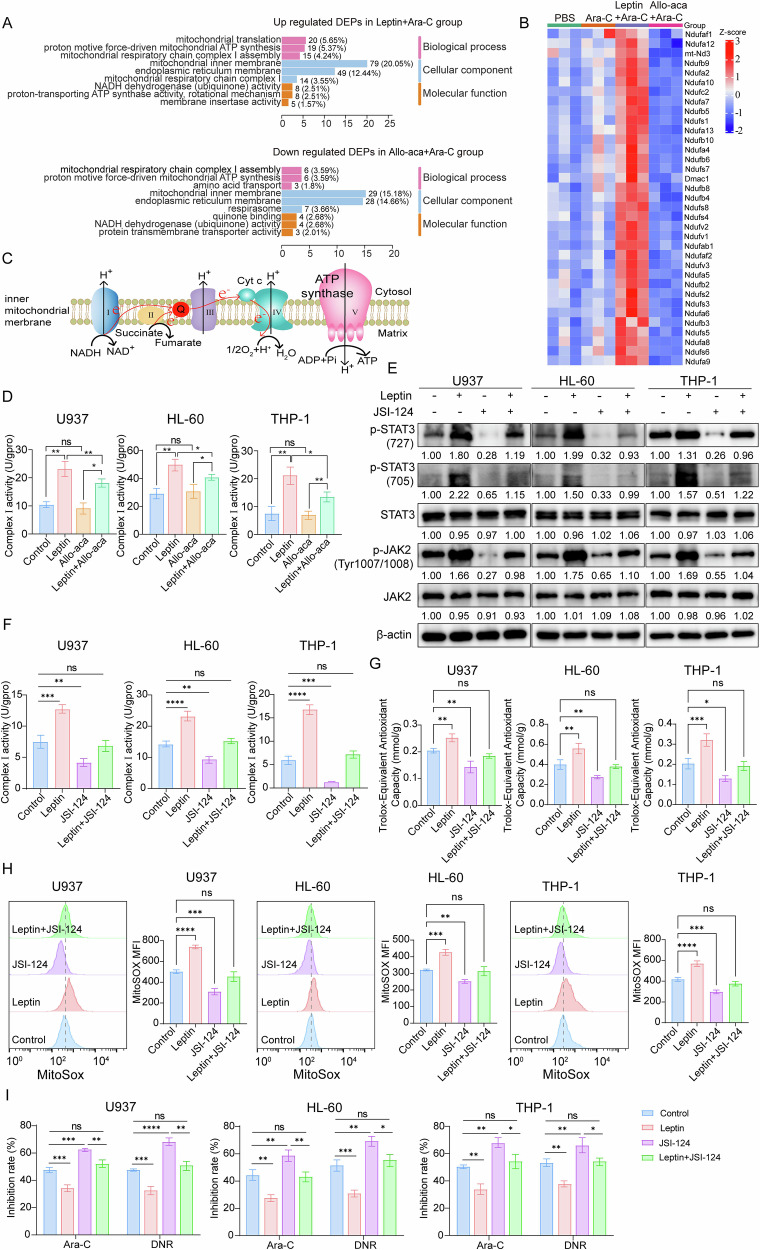
JAK2/STAT3–dependent activation of complex I links leptin to mtROS-driven antioxidant defense. **A** Gene Ontology (GO) terms enrichment analysis of up-regulated DEPs in the leptin + Ara-C group (upper) and down-regulated DEPs in the Allo-aca + Ara-C group (lower). **B** Heatmap analysis of mitochondrial complex I structural subunit protein expression in MLL-AF9 leukemia cells. **C** Structural model of mitochondrial respiratory chain complexes. **D** Comparison of complex I activity in AML cells treated with leptin, Allo-aca, or a combination. **E** Western blot analysis of p-STAT3 (727), p-STAT3 (705), STAT3, p-JAK2 (Tyr1007/1008) and JAK2 expression in AML cells under indicated treatments. β-actin served as a loading control. Rescue experiments of complex I activity **F**, total antioxidant capacity **G** and mtROS production **H** in AML cells. **I** CCK-8 analysis of the chemosensitivity in AML cells treated with leptin, JSI-124 (JAK2/STAT3 inhibitor), or leptin + JSI-124. Data are presented as mean ± SD **D, F–I**. Significance differences were determined by two-tailed unpaired t test **D, I**, or one-way ANOVA with Dunnett’s multiple comparisons test **F–H**. ns, not significant, *p < 0.05, **p < 0.01, ***p < 0.001, ****p < 0.0001.

To clarify the mechanistic basis of leptin-JAK2-STAT3 signaling in metabolic-redox adaptation program, we performed gene-ontology (GO) analysis of proteins up-regulated by leptin + Ara-C and down-regulated by Allo-aca + Ara-C. The results highlighted that DEPs were significantly enriched in ATP synthesis, mitochondrial respiratory chain complex I assembly, and NADH-dehydrogenase activity (Fig. 7A). Indeed, multiple complex I subunits were up-regulated by leptin, suggesting that complex I functionality may be pivotal for leptin-mediated OXPHOS regulation and mtROS production (Fig. 7B). Mitochondrial respiratory chain is composed of five enzyme complexes (complexes I–V), the directional flow of electrons through complex I produce ROS (Fig. 7C). Functional assays further confirmed that leptin selectively boosted complex I activity, whereas left other respiratory complexes unchanged and Allo-aca blunted this increase (Fig. 7D; Fig. S9).

We then employed the JAK2/STAT3 selective inhibitor JSI-124 to examine whether leptin-mediated complex I activation requires JAK2/STAT3 signaling. The findings revealed that leptin reversed JSI-124-induced suppression of p-JAK2 and p-STAT3 (Y705/S727) phosphorylation (Fig. 7E). And blocking JAK2/STAT3 suppressed complex I activity, mtROS production, and antioxidant capacity, while leptin fully rescued each phenotype (Fig. 7F–H). Crucially, JSI-124 restored chemosensitivity in leptin-treated cells (Fig. 7I). Thus, leptin enhances mtROS-dependent antioxidant buffering chiefly by activating complex I through a LEPR-JAK2-STAT3 axis, identifying this signaling node as a tractable target for overcoming chemoresistance in AML.

## Discussion

This study positions marrow adipokines—specifically leptin—and their cognate receptor LEPR as central modulators of chemotherapeutic response in AML. Earlier work showed that LEPR is expressed on AML blasts and that leptin can influence proliferation in vitro or angiogenesis in vivo [33, 34]. However, the impact of leptin-LEPR signaling on AML chemosensitivity remained unexplored. We now significantly extend those observations by demonstrating that elevated marrow leptin levels and blast-cell LEPR expression independently forecast induction failure and poor overall survival, and that leptin–LEPR engagement activates a JAK2/STAT3 → complex I axis that orchestrates a metabolic-redox adaptation program characterized by increased mtROS production, amplified antioxidant defenses, and consequent blunts chemotherapeutic efficacy. Collectively, these findings provide novel insights into leptin–LEPR signaling, positioning it as both a prognostic biomarker and therapeutic target in AML.

While this study focuses on cell-autonomous mechanisms in AML, the bone marrow microenvironment is well established as a critical regulator of the disease and its treatment response. In light of this consensus, bone marrow stromal components—notably adipocytes, mesenchymal stromal cells, and osteoblasts—are known to promote AML chemoresistance through metabolic support, adhesion signaling, and niche remodeling [35–37]. Our findings now identify leptin, which is abundantly secreted by these stromal cells [38–40], as a key mediator of this stroma-induced chemoresistance. To rule out autocrine leptin secretion by AML cells as a confounding factor, we confirmed the absence of leptin in culture supernatants (data not shown). Beyond its direct role, leptin may also indirectly influence chemosensitivity by promoting endothelial cell proliferation, migration, and angiogenesis [41], and driving M1 macrophage polarization to modulate immune responses [42]. Future work employing co-culture and 3D models will be essential to fully elucidate these stroma-dependent mechanisms and to evaluate the therapeutic potential of LEPR antagonism (e.g., using Allo-aca) in disrupting microenvironmental support.

Adipocytes constitute a substantial proportion of the tumor microenvironment and provide both metabolic substrates and paracrine signals to cancer cells [43]. Leptin signaling has been tied to drive multiple pro-tumorigenic effects across malignancies, with particularly well-established roles in mediating chemoresistance. In breast cancer, Wang et al. demonstrated that leptin/JAK/STAT3 signaling drives FAO, thereby promoting cancer stemness and therapy resistance [13]. Similarly, in multiple myeloma, leptin activates AKT/STAT3 pathways to stimulate proliferation and reduce chemotherapy efficacy [44]. Recent work in gallbladder cancer further shows that leptin-induced STAT3 phosphorylation fosters mitochondrial fusion and drug resistance via the CEBPD/MCL1 axis [14]. The role of leptin in colorectal cancer, however, appears more complex and context-dependent. While Suman et al. proposed a mechanism for leptin in promoting radiation-associated carcinogenesis [45], the findings of Aparicio et al. showed in vitro mitogenicity without significant tumor-promoting effects in vivo [46]. Our data reveal a paradigm in which leptin, while not accelerating basal AML progression per se, significantly compromises the therapeutic efficacy of Ara-C in vivo. This effect is corroborated by our in vitro data, which show that leptin alone has minimal impact on baseline proliferation (Fig. S1C), whereas co-treatment with Ara-C consistently attenuates drug-induced growth inhibition. These results indicate that leptin-induced reprogramming of mitochondrial metabolism plays a negligible role in AML growth but is critical for chemoresistance. Specifically, exogenous leptin elevates, whereas the LEPR antagonist Allo-aca lowers, complex I activity and mtROS links stromal signaling directly to redox adaptation—a recognized determinant of drug resistance [20–25, 47–49]. Therapeutically, LEPR blockade or complex I inhibition could therefore decouple adipokine-mediated support of blast cell survival.

Our data identify the canonical leptin-LEPR/JAK2/STAT3 cascade as the fulcrum of leptin-driven chemoresistance in AML, providing a mechanistic explanation for the inferior outcomes observed in patients with high marrow leptin. Clinical and experimental evidence links LEPR overexpression to tumor progression in diverse cancers [50–54], and we likewise found that blasts from high-leptin AML patients express markedly more LEPR than those from low-leptin patients, indicating a tight correlation between stromal leptin and receptor abundance. In vitro, leptin further amplified LEPR levels, consistent with earlier reports [51, 55]. However, CRISPR/Cas9-mediated LEPR ablation profoundly attenuated JAK2/STAT3 signaling, thereby restoring chemosensitivity in AML. Collectively, our study broadens the functional repertoire of leptin-LEPR signaling in AML, underscoring receptor abundance—as a key determinant of redox rewiring and drug response.

The antioxidant defense system comprises enzymatic, non-enzymatic components, and specialized transcription factors with redox-regulating functions [56–58]. In contrast to reported findings that PEITC exerts antileukemic activity by inhibiting mitochondrial respiration and rapidly depleting mitochondrial GSH [30], our data reveal that while PEITC treatment effectively reduces GSH levels, the consequent elevation in mtROS paradoxically stimulates enhanced antioxidant activity, ultimately compromises drug response in AML cells. This effect may be attributed to PEITC-induced upregulation of Nrf2-regulated antioxidant enzymes—including γ-glutamylcysteine-synthetase (γGCS), HO-1, NQO1, and glutathione S-transferase (GST)—which collectively enhance cellular oxidative stress defense mechanisms [59–61]. Our study identifies a novel resistance mechanism whereby PEITC triggers compensatory antioxidant responses that limit AML therapeutic efficacy.

Contrary to their cytotoxic potential, ROS frequently serve as mediators of adaptive oxidative stress responses in malignancies, enabling evasion of apoptosis and acquisition of therapeutic resistance [62, 63]. ROS-mediated chemoresistance in cancer cells arises through coordinated activation of redox-sensitive transcription factors (NF-κB, Nrf2, c-Jun and HIF-1α), which concomitantly bolster antioxidant defenses and upregulate pro-survival protein networks [64, 65]. Mitochondrial electron transfer chain (ETC)-derived ROS are primary drivers of cellular oxidative stress during respiration. At several sites along the respiratory chain—including complex I, complex Ⅲ, and α-ketoglutarate dehydrogenase—electrons can leak onto molecular oxygen, forming superoxide radicals (O₂·⁻) [66, 67]. Complex I, in particular, is a dominant mitochondrial ROS source and plays a key regulator of metabolic homeostasis [68, 69], and as we show here, a convergence point for leptin-LEPR signaling. By boosting complex I activity, leptin raises mtROS that paradoxically triggers a counter-balancing antioxidant surge—principally through up-regulation of Nrf2 targets, glutathione metabolism and ROS-detoxifying enzymes. This adaptive loop mirrors observations that elevated Nrf2 levels, *FLT3-ITD* or stromal mitochondrial transfer fortify AML redox buffering [24, 70–72]. Pharmacological complex I inhibition (e.g., IACS-010759) is already under clinical evaluation in AML; integrating LEPR antagonism or JAK2/STAT3 blockade could synergistically collapse this metabolic shield.

Our work highlights adipose–tumor crosstalk as a generalizable vulnerability. Because marrow adiposity increases with age—mirroring AML incidence—systemic metabolic states (obesity, fasting, cachexia) might modulate drug sensitivity via leptin dynamics. Prospective studies correlating body mass index, marrow adipocyte content, and treatment outcome will be informative. Mechanistically, how LEPR up-regulation is transcriptionally or post-transcriptionally controlled in blasts, and whether specific receptor isoforms harbor distinct signaling properties, remain open questions. Finally, combining LEPR blockade with agents that generate ROS (e.g., venetoclax/Azacitidine or menin inhibitors) could unmask synthetic-lethal interactions and merits pre-clinical testing.

In summary, we identify leptin–LEPR signaling as a marrow-derived driver of mitochondrial rewiring that enforces an mtROS-dependent antioxidant shield and promotes chemotherapy resistance in AML. Targeting LEPR or its downstream JAK2/STAT3–complex I node offers a promising strategy to dismantle this protective circuit and improve treatment outcomes.

## Supplementary information

Supplementary materials

Supplementary material

## Data Availability

The mass spectrometry proteomics data have been deposited to the ProteomeXchange Consortium (https://proteomecentral.proteomexchange.org) via the iProX partner repository with the dataset identifier PXD066693. The data that support the findings of this study are available from corresponding author upon reasonable request.
