# Supplementary material for: Marrow leptin-LEPR signaling rewires mitochondrial oxidative metabolism to confer chemoresistance in acute myeloid leukemia
Source: Cell Death Dis. 2026 Feb 23;17(1):249. doi: 10.1038/s41419-026-08528-0 (PMC12966444; doi:10.1038/s41419-026-08528-0)
Supplement: Supplementary file 1 — Supplementary materials [file 41419_2026_8528_MOESM1_ESM.doc]

**Supplementary Materials**

**Marrow leptin-LEPR signaling rewires mitochondrial oxidative metabolism to confer chemoresistance in acute myeloid leukemia**

Xinai Liao, Wei Dai, Xiaolin Xu, Danni Cai, Maoqing Tan, Zukai Wang, Yanrong Huang, Diyu Hou, Jingru Liu, Liuhuan Wang, Jin Wang, Xiaoting Wang, Shuxia Zhang, Xinjian Lin, and Huifang Huang

**
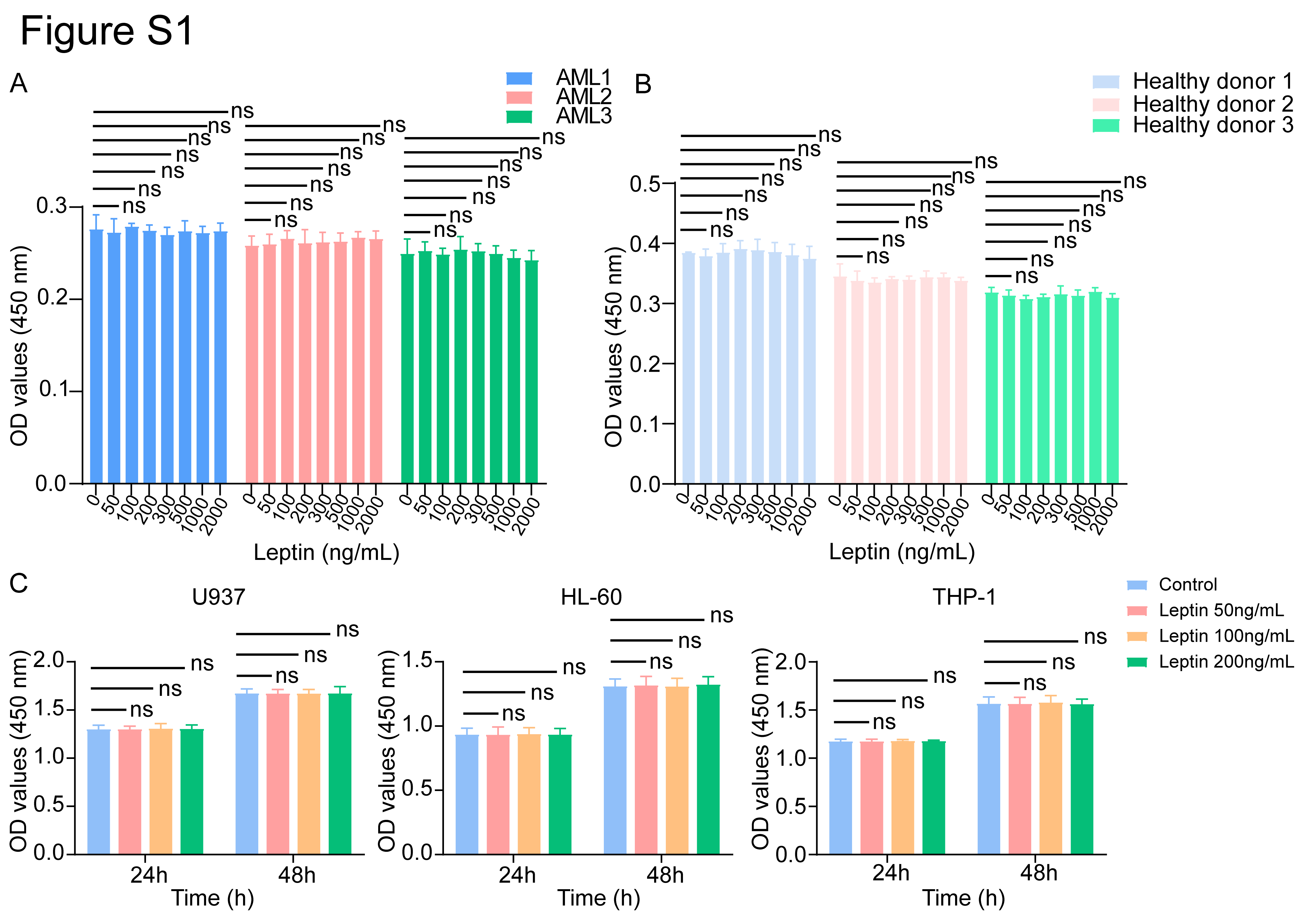
**

**Figure S1. Leptin has no effect on cell proliferation**

(A-B) CCK-8 assays were performed to assess the proliferation of patient-derived AML blasts (A) and healthy donor peripheral blood mononuclear cells (B) after 24-hour treatment with leptin at the indicated concentrations (n = 3). (C) CCK-8 assay evaluating the effects of graded leptin concentrations on AML cell growth following 24 h or 48 h treatment. Data are presented as mean ± SD. Statistical significance was determined by one-way ANOVA with Dunnett's multiple comparisons test. ns, not significant.

**
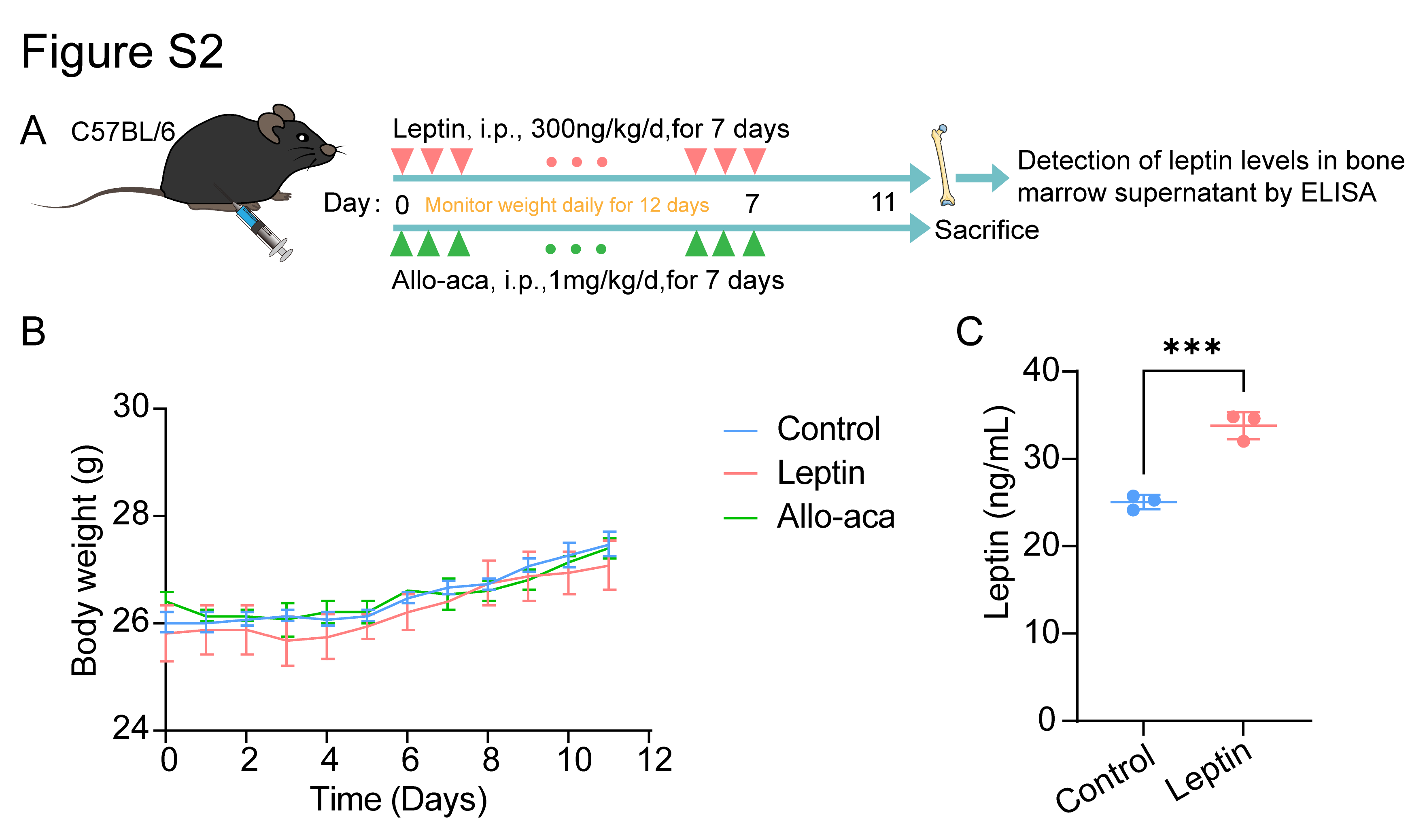
**

**Figure S2.** **The dose chosen of leptin in mouse models**

(A) Experimental design. (B) Body weight monitoring of mice treated with leptin or Allo-aca (n = 3 mice per group). (C) Leptin levels in BM supernatants of mice after intraperitoneal leptin administration (300ng/kg/d, for 7 days) (n = 3 mice per group). Data are presented as mean ± SD (B and C). Significance differences were determined by one-way ANOVA with Dunnett's multiple comparisons test (C). ****p* < 0.001.

**
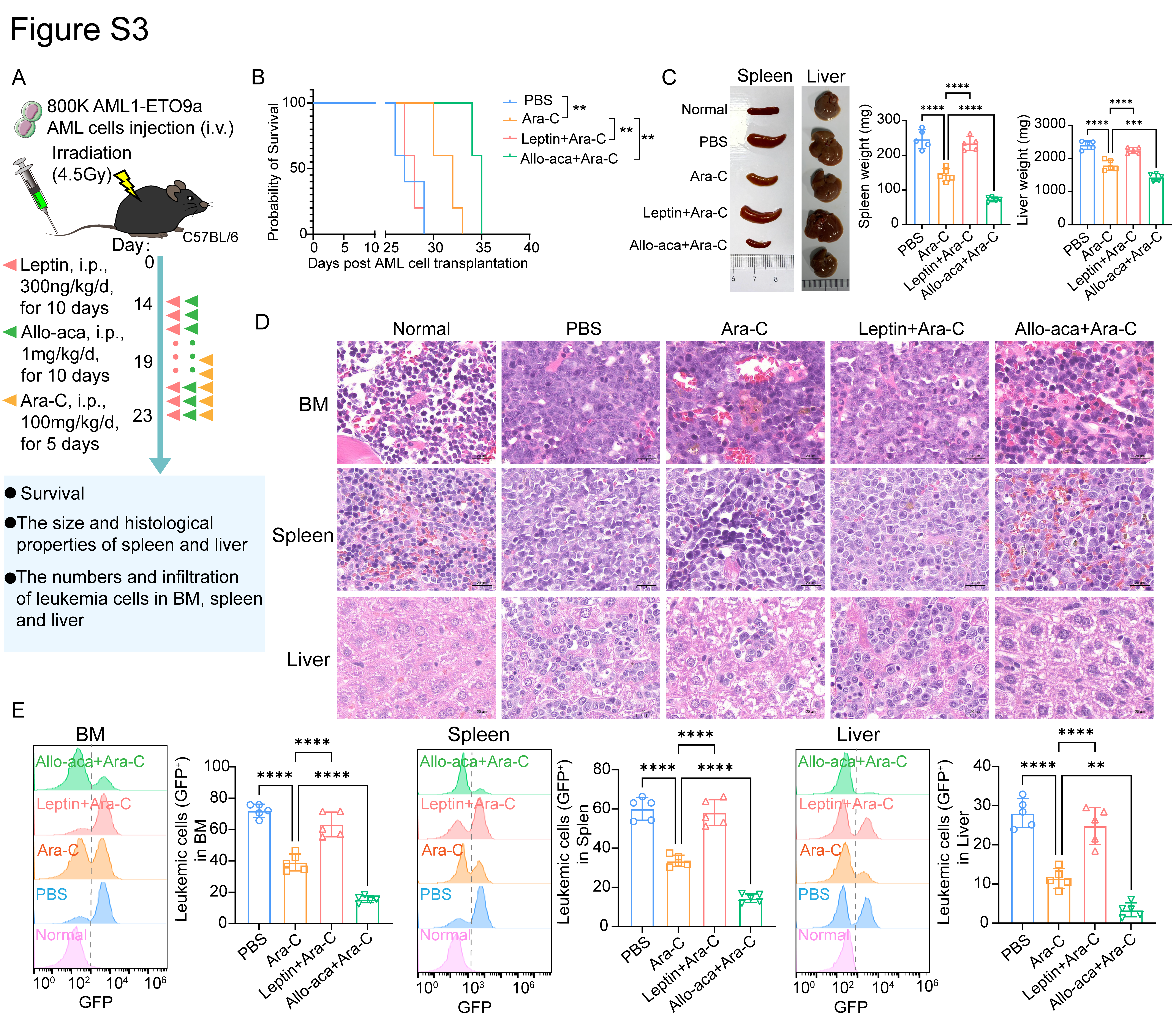
**

**Figure S3. Leptin–LEPR signaling limits cytarabine efficacy in AML mouse models (AML1-ETO9a)**

(A) Schematic of the experimental design evaluating leptin’s impact on chemotherapy response in AML1-ETO9a-driven AML mice. (B) Kaplan–Meier survival curves (n = 5 per group). (C) Representative spleen and liver images with organ weight comparisons (n = 5 per group). (D) Representative H&E staining of bone marrow (BM), spleen, and liver (scale bars, 20 μm). (E) Flow cytometry histograms (left) and quantification (right) of GFP⁺ AML cells in BM, spleen, and liver (n = 5 per group). Data are mean ± SD (C, E). Statistical significance was determined by log-rank test (B) and one-way ANOVA with Dunnett’s multiple comparisons (C, E). ***p* < 0.01, ****p* < 0.001, *****p* < 0.0001.

**
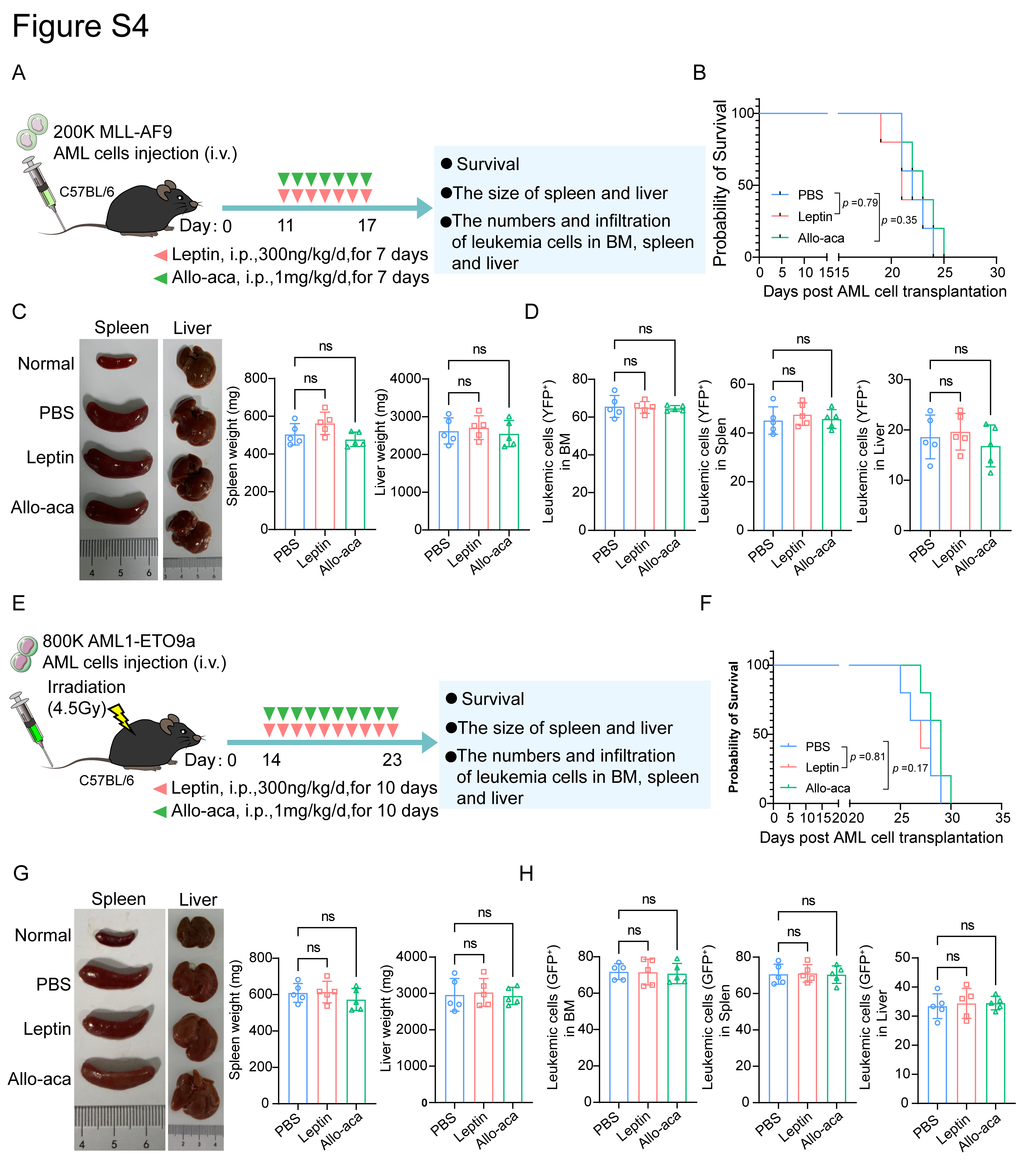
**

**Figure S4. Leptin–LEPR signaling does not alter basal leukemia growth**

(A) Schematic diagram for evaluating the effect of leptin on leukemia progression in MLL-AF9-driven AML mice. (B) Kaplan-Meier survival curves (n = 5 mice per group). (C) Representative images and weight comparisons of spleen and liver in MLL-AF9-driven mice (n = 5 mice per group). (D) Comparison of YFP^+^ leukemia cell percentages in BM, spleen and liver from MLL-AF9 AML mice (n = 5 mice per group). (E) Schematic diagram of AML1-ETO9a-driven AML mice. (F) Kaplan-Meier survival curves (n = 5 mice per group). (G) Representative pictures and weight comparisons of spleen and liver. (n = 5 mice per group). (H) Quantification of the percentage of GFP^+^ leukemia cells in BM, spleen and liver from AML1-ETO9a AML mice (n = 5 mice per group). Data are presented as mean ± SD (C, D, G and H). Statistical significance was determined by log-rank test (B and F), or one-way ANOVA with Dunnett's multiple comparisons test (C, D, G and H). ns, not significant.


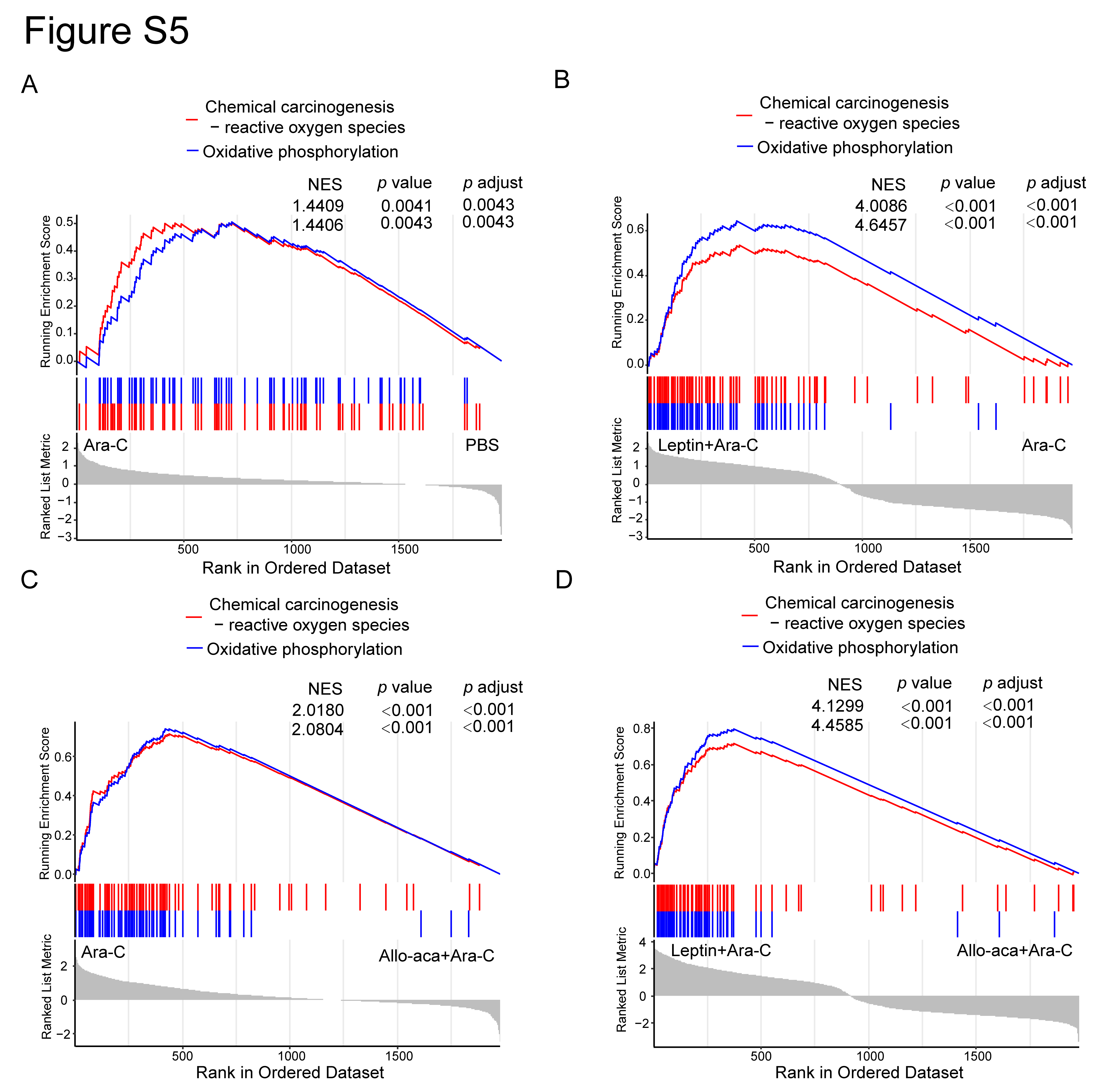


**Figure S5. GSEA analysis**

(A-D) GSEA analysis of OXPHOS (Ko00190) and ROS (Ko05208) pathway in MLL-AF9 leukemia cells among the four groups: (A) Ara-C vs PBS; (B) Leptin+Ara-C vs Ara-C; (C) Ara-C vs Allo-aca+Ara-C; (D) Leptin+Ara-C vs Allo-aca+Ara-C.


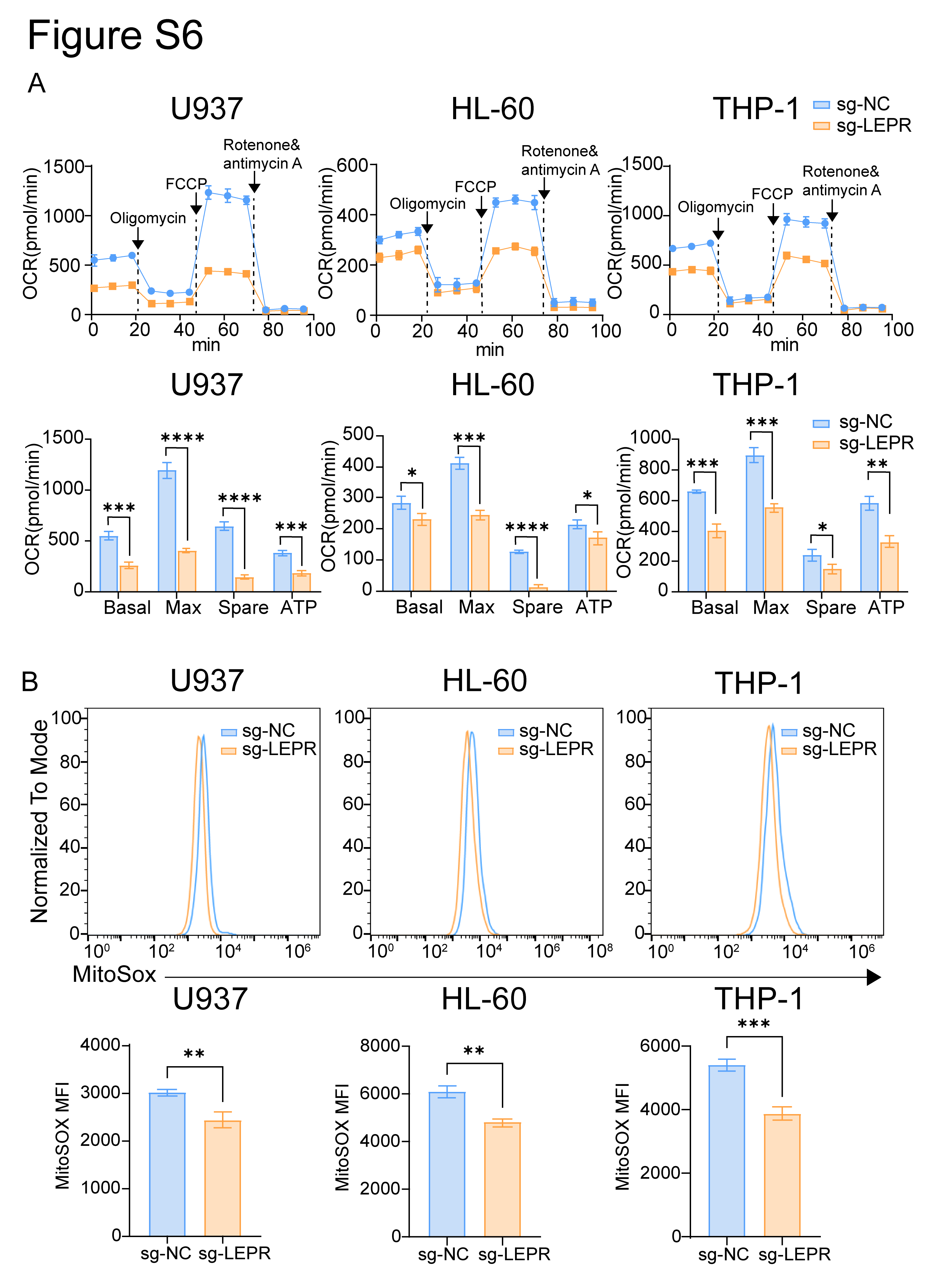


**Figure S6. Impact of *LEPR* knockout on** **OXPHOS levels and mtROS production in AML cells**

(A) Oxygen consumption rate (OCR) traces (top) and quantification of basal respiration, maximal respiration, spare respiratory capacity, and ATP-linked respiration (bottom) in *LEPR*-knockout versus control AML cells. (B) Flow-cytometry histograms (top) and mean fluorescence intensity (MFI) quantification (bottom) of mtROS in *LEPR*-knockout versus control AML cells. Data are presented as mean ± SD. Statistical significance was determined by two-tailed unpaired *t* tests. **p* < 0.05, ***p* < 0.01, ****p* < 0.001, *****p* < 0.0001.


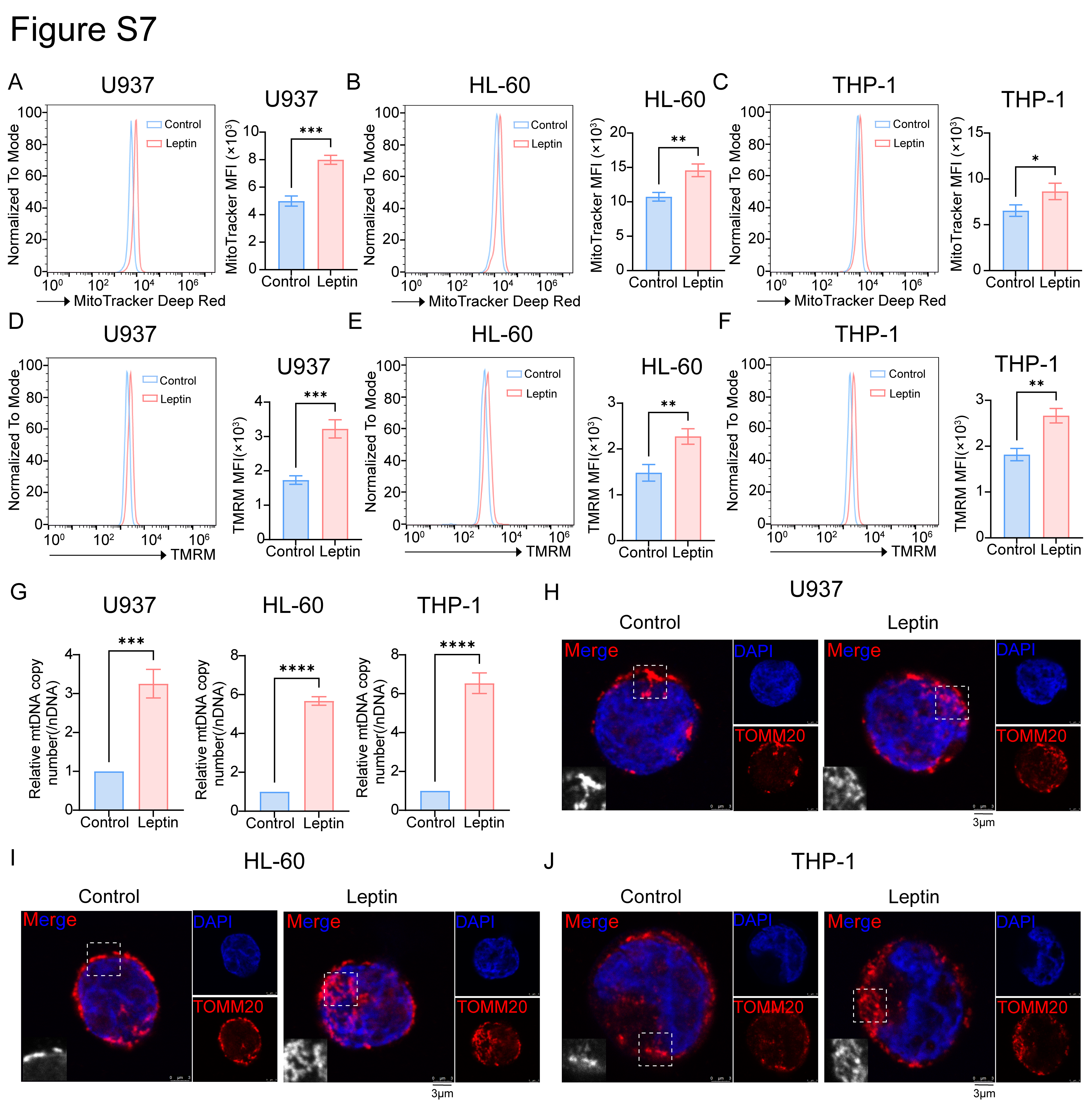


**Figure S7. Leptin promotes mitochondrial biogenesis in AML cells**

(A-C) Representative FCM profiles (left) and comparison of MFI (right) in U937 (A), HL-60 (B) and THP-1 (C) cells stained with MitoTracker Deep Red after leptin treatment. (D-F) Representative FCM profiles (left) and MFI statistical analysis (right) of membrane potential in leptin-treated U937 cells (D), HL-60 cells (E) and THP-1 cells (F). (G) Relative quantification of mtDNA copy numbers in AML cells treated with or without leptin. (H) Representative confocal images for immunofluorescence analysis of mitochondrial morphology (TOMM20, red) in U937 cells (H), HL-60 cells (I) and THP-1 cells (J) (scale bars: 3 μm). Data are presented as mean ± SD (A-G) and were analyzed by one-way ANOVA with Dunnett's multiple comparisons test. **p* < 0.05, ***p* < 0.01, ****p* < 0.001, *****p* < 0.0001.

**Figure S8. Leptin/LEPR impacts JAK2-STAT3 signaling *in vivo* and *in vitro***

(A and B) Representative images for immunofluorescence analysis of LEPR (A, yellow), p-STAT3 (705) (A, red), p-JAK2 (B, yellow) and p-STAT3 (727) (B, red) expression in BM leukemia cells of MLL-AF9 AML mice (scale bars: 100 μm). (C and D) Representative immunofluorescence images showing expression of LEPR (C, yellow), p-STAT3 (705) (C, red), p-JAK2 (D, yellow) and p-STAT3 (727) (D, red) in BM leukemia cells from AML1-ETO9a AML mice (scale bars: 100 μm). (E) Quantification of LEPR, p-JAK2, p-STAT3 (705), p-STAT3 (727) expression presented in (A and B) (n = 5 mice per group). (F) Comparison of LEPR, p-JAK2, p-STAT3 (705), p-STAT3 (727) expression presented in (C and D) (n = 5 mice per group). (G and H) Western blot of LEPR/JAK2/STAT3 signaling was performed in AML cells treated with leptin or *LEPR*-knockout*.* β-actin served as a loading control.​ Data are presented as mean ± SD (E and F) and were analyzed by one-way ANOVA with Dunnett's multiple comparisons test. ns, not significant, ***p* < 0.01, ****p* < 0.001, *****p* < 0.0001.


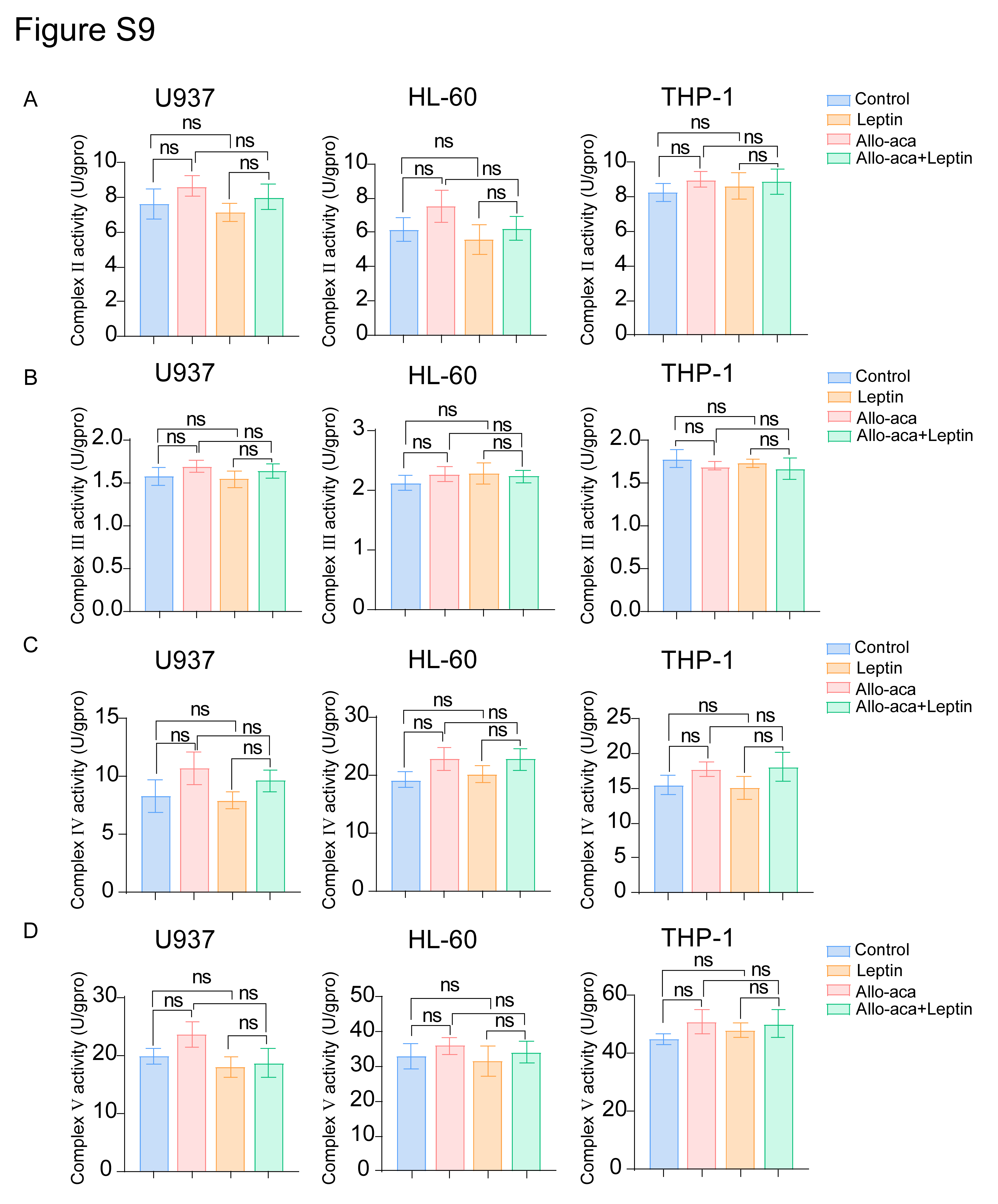


**Figure S9. Leptin has no significant effect on complex Ⅱ-Ⅴ activity**

(A-D) Comparison the activity of complex Ⅱ (A), complex Ⅲ (B), complex Ⅳ (C) and complex Ⅴ (D). Data are presented as mean ± SD and were analyzed by two-tailed unpaired t test. ns, not significant.

**
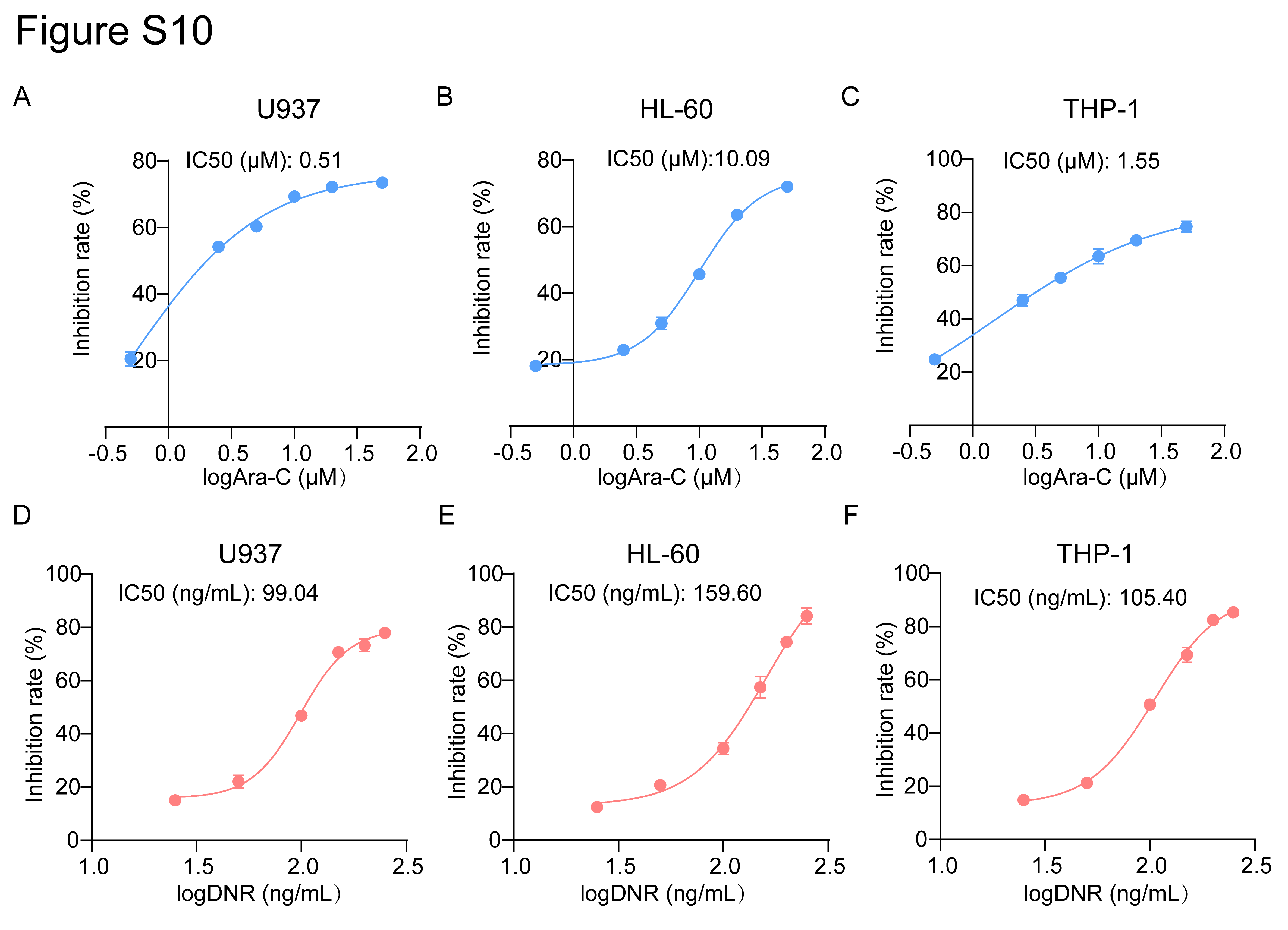
**

**Figure S10. IC50 of chemotherapy drugs in human AML cell lines**

(A-C) CCK-8 analysis of Ara-C IC50 in U937 cells (A), HL-60 cells (B) and THP-1 cells (C). (D-F) IC50 of DNR in U937 cells (D), HL-60 cells (E) and THP-1 cells (F).

**Table S1. Characteristics of newly diagnosed AML patients in this study**

| **UPN** | **Age /Sex** | **FAB** | **Pretreatment**  **BM blasts^a^ (%)** | **Post-treatment**  **BM blasts^b^ (%)** | **Blast cells** **clearance rate (%)** | **Risks^c^**  **(ELN 2022)** | **Chemotherapy efficacy^d^** | **Leptin**  **(ng/mL)** |
| --- | --- | --- | --- | --- | --- | --- | --- | --- |
| 1 | 23/F | M5 | 36.0 | 0.5 | 98.61 | Adverse | CRi | 1.86 |
| 2 | 54/M | M5 | 45.5 | 3.5 | 92.31 | Favorable | CRi | 1.77 |
| 3 | 27/M | M5 | 10.0 | 0.5 | 95.00 | Intermediate | CRi | 4.94 |
| 4 | 63/M | M5 | 32.5 | 1.0 | 96.92 | Adverse | CR | 7.91 |
| 5 | 43/F | M5 | 45.0 | 2.0 | 95.56 | Intermediate | CR | 4.53 |
| 6 | 33/M | M1 | 89.0 | 47.5 | 46.63 | Intermediate | NR | 9.61 |
| 7 | 52/M | M2 | 40.5 | 10.0 | 75.31 | Adverse | CR | 10.04 |
| 8 | 58/F | M5 | 5.0 | 3.5 | 30.00 | Adverse | CRi | 0.64 |
| 9 | 56/M | M2 | 39.0 | 16.0 | 58.97 | Favorable | NR | 6.52 |
| 10 | 19/F | M5 | 45.0 | 2.0 | 95.56 | Adverse | CRi | 8.99 |
| 11 | 43/F | M2 | 81.0 | 0.0 | 100.00 | Favorable | CR | 0.35 |
| 12 | 20/M | M2 | 84.0 | 0.5 | 99.40 | Favorable | CR | 4.96 |
| 13 | 67/F | M5 | 4.0 | 0.5 | 87.50 | Adverse | CR | 7.15 |
| 14 | 50/M | M2 | 80.0 | 2.0 | 97.50 | Favorable | CR | 5.64 |
| 15 | 54/F | M5 | 17.5 | 1.5 | 91.43 | Intermediate | CRi | 5.09 |
| 16 | 47/M | M2 | 77.5 | 1.0 | 98.71 | Adverse | CRh | 4.84 |
| 17 | 46/M | M5 | 29.5 | 29.5 | 0.00 | Favorable | NR | 6.97 |
| 18 | 39/M | M5 | 46.0 | 4.0 | 91.30 | Favorable | CRi | 2.70 |
| 19 | 59/M | M2 | 42.0 | 10.0 | 76.19 | Adverse | CR | 10.48 |
| 20 | 19/F | M5 | 50.0 | 0.0 | 100.00 | Intermediate | CRh | 7.15 |
| 21 | 30/F | M5 | 36.0 | 1.0 | 97.22 | Adverse | CRi | 2.65 |
| 22 | 23/F | M5 | 51.0 | 0.5 | 99.02 | Intermediate | CRh | 9.31 |
| 23 | 79/M | M1 | 96.0 | 11.5 | 88.02 | Intermediate | PR | 2.04 |
| 24 | 68/M | M2 | 72.5 | 38.5 | 46.90 | Adverse | NR | 12.04 |
| 25 | 64/M | M5 | 30.0 | 0.5 | 98.33 | Intermediate | CR | 1.40 |
| 26 | 19/M | M2 | 41.0 | 1.0 | 97.56 | Favorable | CR | 3.67 |
| 27 | 61/F | M5 | 18.0 | 0.0 | 100.00 | Intermediate | CR | 1.05 |
| 28 | 57/F | M5 | 5.0 | 4.0 | 20.00 | Adverse | CRi | 0.73 |
| 29 | 57/F | M5 | 25.0 | 0.5 | 98.00 | Favorable | CRi | 1.51 |
| 30 | 40/M | M5 | 31.5 | 0.5 | 98.41 | Favorable | CR | 4.72 |
| 31 | 49/M | M2 | 40.0 | 15.0 | 62.50 | Favorable | NR | 6.92 |
| 32 | 55/F | M2 | 80.0 | 0.5 | 99.38 | Favorable | CR | 0.73 |
| 33 | 85/F | M1 | 96.0 | 11.0 | 88.54 | Intermediate | PR | 2.35 |
| 34 | 48/F | M5 | 12.5 | 0.0 | 100.00 | Adverse | CR | 8.55 |
| 35 | 51/M | M2 | 46.0 | 2.5 | 94.57 | Intermediate | CRi | 10.76 |
| 36 | 37/M | M2 | 30.5 | 2.0 | 93.44 | Favorable | CRi | 5.46 |
| 37 | 19/M | M4 | 32.5 | 1.0 | 96.92 | Favorable | CR | 2.62 |
| 38 | 36/M | M5 | 46.0 | 0.5 | 98.91 | Intermediate | CRi | 1.41 |
| 39 | 60/F | M1 | 77.0 | 0.0 | 100.00 | Intermediate | CR | 3.13 |
| 40 | 68/F | M5 | 12.0 | 10.0 | 16.67 | Adverse | NR | 10.83 |
| 41 | 49/M | M5 | 26.0 | 6.0 | 76.92 | Adverse | CR | 7.08 |

**Table S1 (continued)**

| **UPN** | **Age /Sex** | **FAB** | **Pretreatment**  **BM blasts^a^ (%)** | **Post-treatment**  **BM blasts^b^ (%)** | **Blast cells** **clearance rate (%)** | **Risks^c^**  **(ELN 2022)** | **Chemotherapy efficacy^d^** | **Leptin**  **(ng/mL)** |
| --- | --- | --- | --- | --- | --- | --- | --- | --- |
| 42 | 21/M | M2 | 73.5 | 0.0 | 100.00 | Favorable | CR | 1.86 |
| 43 | 64/F | M5 | 25.0 | 1.5 | 94.00 | Intermediate | CR | 5.18 |
| 44 | 57/F | M5 | 3.0 | 0.5 | 83.33 | Adverse | CR | 1.41 |
| 45 | 29/M | M5 | 5.5 | 0.0 | 100.00 | Intermediate | CRi | 3.09 |
| 46 | 43/M | M2 | 36.0 | 1.5 | 95.83 | Favorable | CR | 1.06 |
| 47 | 42/M | M5 | 12.5 | 12.5 | 0.00 | Favorable | NR | 6.77 |
| 48 | 32/F | M5 | 33.0 | 0.0 | 100.00 | Adverse | CR | 7.90 |
| 49 | 30/M | M2 | 53.5 | 10.0 | 81.31 | Favorable | CR | 8.44 |
| 50 | 60/F | M2 | 19.0 | 8.5 | 55.26 | Adverse | NR | 10.34 |
| 51 | 64/M | M5 | 11.5 | 3.0 | 73.91 | Adverse | CR | 9.56 |
| 52 | 71/M | M5 | 42.0 | 2.0 | 95.24 | Adverse | CR | 9.12 |
| 53 | 55/M | M2 | 78.5 | 1.5 | 98.09 | Favorable | CR | 1.00 |
| 54 | 81/F | M1 | 95.5 | 12.0 | 87.43 | Intermediate | PR | 1.69 |
| 55 | 26/F | M5 | 49.0 | 1.0 | 97.96 | Adverse | CR | 6.55 |
| 56 | 79/M | M1 | 95.0 | 10.5 | 88.95 | Intermediate | PR | 1.85 |
| 57 | 66/F | M2 | 82.0 | 4.0 | 95.12 | Intermediate | CRi | 2.09 |
| 58 | 68/F | M1 | 91.0 | 1.5 | 98.35 | Intermediate | CR | 7.47 |
| 59 | 39/M | M2 | 31.0 | 2.5 | 91.94 | Favorable | CRi | 5.48 |
| 60 | 56/M | M5 | 23.0 | 0.0 | 100.00 | Favorable | CR | 4.81 |
| 61 | 46/F | M5 | 41.0 | 10.0 | 75.61 | Intermediate | CR | 7.44 |
| 62 | 83/M | M1 | 94.0 | 10.0 | 89.36 | Intermediate | PR | 2.88 |
| 63 | 57/M | M5 | 17.5 | 4.5 | 74.29 | Intermediate | NR | 8.99 |
| 64 | 69/F | M2 | 21.0 | 2.0 | 90.48 | Adverse | CR | 9.37 |
| 65 | 47/F | M5 | 13.0 | 0.5 | 96.15 | Adverse | CR | 9.19 |
| 66 | 64/F | M5 | 18.0 | 2.0 | 88.89 | Intermediate | CRi | 3.50 |
| 67 | 39/F | M2 | 79.0 | 1.5 | 98.10 | Favorable | CR | 0.73 |
| 68 | 26/F | M5 | 22.5 | 5.0 | 77.78 | Favorable | CR | 8.69 |
| 69 | 34/F | M5 | 14.0 | 0.5 | 96.43 | Intermediate | CRi | 5.94 |
| 70 | 67/F | M5 | 42.5 | 7.0 | 83.53 | Adverse | NR | 10.04 |
| 71 | 20/F | M5 | 49.0 | 0.0 | 100.00 | Favorable | CRh | 3.71 |
| 72 | 42/F | M5 | 30.0 | 0.0 | 100.00 | Favorable | CR | 3.98 |
| 73 | 61/M | M5 | 43.5 | 0.0 | 100.00 | Intermediate | CRh | 8.89 |
| 74 | 51/M | M5 | 19.0 | 5.0 | 73.68 | Intermediate | NR | 10.25 |
| 75 | 49/M | M2 | 80.0 | 5.0 | 93.75 | Adverse | CRh | 7.41 |
| 76 | 34/M | M1 | 89.5 | 48.0 | 46.37 | Intermediate | NR | 9.03 |
| 77 | 27/M | M2 | 27.0 | 6.0 | 77.78 | Adverse | CR | 6.70 |
| 78 | 66/F | M5 | 31.0 | 2.0 | 93.55 | Intermediate | CRh | 5.90 |
| 79 | 45/M | M5 | 46.5 | 12.0 | 74.19 | Adverse | CRi | 8.53 |
| 80 | 45/M | M5 | 30.0 | 30.0 | 0.00 | Favorable | NR | 6.54 |
| 81 | 48/F | M2 | 63.0 | 1.0 | 98.41 | Favorable | CRh | 0.49 |
| 82 | 30/M | M0 | 73.0 | 15.0 | 79.45 | Adverse | CRi | 10.12 |

**Table S1 (continued)**

| **UPN** | **Age /Sex** | **FAB** | **Pretreatment**  **BM blasts^a^ (%)** | **Post-treatment**  **BM blasts^b^ (%)** | **Blast cells** **clearance rate (%)** | **Risks^c^**  **(ELN 2022)** | **Chemotherapy efficacy^d^** | **Leptin**  **(ng/mL)** |
| --- | --- | --- | --- | --- | --- | --- | --- | --- |
| 83 | 38/M | M5 | 57.0 | 1.0 | 98.25 | Intermediate | CRi | 2.00 |
| 84 | 51/M | M2 | 23.5 | 0.0 | 100.00 | Intermediate | CRi | 0.52 |

^a^Proportion of BM blasts at diagnosis. ^b^Proportion of BM blasts at week 4 after initial chemotherapy. ^c^Risk classification followed the 2022 ELN criteria. ^d^ORR (objective response rate) = CR + CRi + PR. BM, bone marrow; FAB, French-American-British classification; ELN 2022, European LeukemiaNet 2022 risk classification; CR, complete remission; Cri, CR with incomplete hematologic recovery; PR, partial remission; NR, non-response.

**Table S2. The sgRNA sequences target the *LEPR* gene**

| sgRNA | Target sequence |
| --- | --- |
| LEPR#1 | GTATTCTTTGAGAGTCCAGC |
| LEPR#2 | CAAACTGGAACATACAGTGC |
| Negative control | CGCTTCCGCGGCCCGTTCAA |

**Table S3. Primer sequences for RT-qPCR**

| Gene  (human) | Forward  (5'-3') | | Reverse  (5'-3') |
| --- | --- | --- | --- |
| *Nrf2* | ACACGGTCCACAGCTCATC | TGTCAATCAAATCCATGTCCTG | |
| *GCLM* | TGTCTTGGAATGCACTGTATCTC | CCCAGTAAGGCTGTAAATGCTC | |
| *GCLC* | AGAGAAGGGGGAAAGGACAAAC | AAGTTATTGTGCAAAGAGCCTGAT | |
| *HMOX1* | CTCAAACCTCCAAAAGCC | TCAAAAACCACCCCAACCC | |
| *NQO1* | ATGTATGACAAAGGACCCTTCC | TCCCTTGCAGAGAGTACATGG | |
| *HO-1* | AACTTTCAGAAGGGCCAGGT | CTGGGCTCTCCTTGTTGC | |
| *CAT* | ACTTTGAGGTCACACATGACATT | CTGAACCCGATTCTCCAGCA | |
| *GPX1* | TGCAACCAGTTTGGGCATCA | ACCGTTCACCTCGCACTTC | |
| *GPX4* | CAGTGAGGCAAGACCGAAGT | CTGCTTCCCGAAGTGGTTAC | |
| *SOD1* | AGGGCATCATCAATTTCGAGC | GCCCACCGTGTTTTCTGGA | |
| *SOD2* | AACCTCAGCCCTAACGGTG | AGCAGCAATTTGTAAGTGTCCC | |
| *GAPDH* | CTGACTTCAACAGCGACACC | TGCTGTAGCCAAATTCGTTGT | |

**Materials and Methods**

**Analysis of association between marrow plasma leptin levels and clinical characteristics**

Patients were stratified into high- or low-leptin groups using the median leptin level (5.471 ng/mL) as the cutoff. Associations between leptin stratification groups (high vs low) and clinical parameters were assessed using chi-squared tests for categorical variables, including sex, age, peripheral WBC count, blast percentage, karyotype, French-American-British (FAB) classification and ELN 2022 risk classification.

**Chemosensitivity assays**

Primary AML-blasts were pretreated with recombinant human leptin (200 ng/mL, R&D Systems) for 24 h before plating (2×10^4^ cells/well in 96-well plates). Following 24-hour Ara-C (100 μM) treatment, cell viability was assessed using CCK-8 assays (Seven Biotechnology, Beijng, China), with leptin maintained throughout the experiment.

For AML cell lines (U937, HL-60 and THP-1), cells were treated for 24 h with IC50 doses of therapeutic agents (determined in Figure S10) and growth inhibition quantified by CCK-8. All the cell lines have been authenticated by short tandem repeat (STR) profiling. Cell lines were treated with agents under standardized conditions: 24 h leptin (200 ng/mL) pretreatment followed by 24 h co-treatment with therapeutic agents (total leptin exposure: 48 h), Allo-aca (300 ng/mL, 24 h), Cucurbitacin I (100 nM, 20 h; MedChemExpress), Mito-TEMPO (10 μM, 20 h; MedChemExpress), PEITC (5 μM, 5 h; Aladdin, Shanghai, China). The absorbance was read using a microplate reader (SpectraMax i3x, Molecular Devices, San Jose, CA, USA) at 450nm. Chemotherapy-induced cytotoxicity under various interventions was assessed by the inhibition rate, calculated as: Inhibition Rate (%) = [(Ac - As) / (Ac - Ab)] × 100%, where Ac, As, and Ab denote the absorbance of the negative control, the test sample, and the background blank (cell-free medium), respectively.

**Flow cytometry analysis**

**(1) Tissue processing and leukemic burden quantification**

Single-cell suspensions were prepared from bone marrow, spleen and liver tissues of mice. After centrifugation, red blood cells were lysed by RBC lysis buffer (Biolegend, San Diego, CA, USA; 15 min on ice). Cells were washed with PBS containing 2% fetal bovine serum (FBS, Gibco, Waltham, MA, USA), and leukemic burden was quantified by FCM (BD Biosciences FACS Celesta, San Jose, CA, USA) based on YFP or GFP expression.

**(2) LEPR expression detection in primary AML cells**

Primary BMMNCs were rapidly thawed from cryopreservation and cultured in a humidified incubator at 37°C, with 5% CO_2_ for 24 h. Following PBS washing, cells were stained with anti-human CD45-APC (BD Biosciences,San Jose, CA, USA, Cat# 561864) and CD34-PerCP-Cy5.5 (BD Biosciences, Cat# 570578) in staining buffer (PBS + 2% FBS) at 4°C for 30 min. Cells were further incubated with anti-human LEPR antibody (Proteintech, Wuhan, Hubei, China, Cat# 20966-1-AP) at 4°C for 30 min and Alexa Fluor 555-conjugated secondary antibody (Beyotime, Nanjing, Jiangsu, China, ) at 37°C for 30 min, followed by FCM analysis.

**(3) Mitochondrial assessment**

Three key mitochondrial parameters were evaluated: mitochondrial mass using MitoTracker Deep Red (Invitrogen), membrane potential via tetramethylrhodamine methyl ester (TMRM, MedChemExpress) and mtROS levels with MitoSOX Red (Invitrogen). For each measurement, cells were incubated with the respective fluorescent probe at 37°C for 30 min under light-protected conditions. Following incubation, cells were washed twice with PBS and immediately analyzed by FCM. In all experiments, live cells were discriminated using Fixable Viability Dye eFluor™ 450 (eBioscience, San Diego, CA, USA), with dead cells excluded from subsequent analysis.

**Western blot analysis**

Total cellular proteins were extracted using ice-cold RIPA lysis buffer (Beyotime) supplemented with phosphatase inhibitor cocktail (MedChemExpress), protease inhibitor cocktail (MedChemExpress) and PMSF (Dingguo). Lysates were centrifuged at 12,000 × g for 15 min at 4°C, and protein concentrations in the supernatants were quantified using the BCA Protein Assay Kit (Thermo Fisher Scientific, Rockford, IL, USA) according to the manufacturer's protocol. Proteins were separated by 10% SDS-PAGE and transferred onto 0.45 μm PVDF membranes (Merck Millipore). Membranes were blocked with 5% (w/v) non-fat dry milk in TBST (Tris-buffered saline with 0.1% Tween-20) for 3 h at room temperature, followed by incubation with primary antibodies overnight at 4°C. The primary antibodies included: STAT3 (Cell Signaling Technology, Cat# 12640S), Phospho-STAT3(Ser727) (Cell Signaling Technology, Cat# 9134S), Phospho-STAT3(Tyr705) (Cell Signaling Technology, Cat# 9145S), JAK2 (Cell Signaling Technology, Cat# 3230S), Phospho-JAK2 (Tyr1007/Tyr1008) (Cell Signaling Technology, Cat# 3771S), LEPR (Proteintech, Cat# 20966-1-AP), Phospho-LEPR(Tyr986) (Immunoway, Cat# YP1805), Phospho-LEPR (Tyr1411) (Immunoway, Cat# YP1200) and β-actin (Beyotime, Cat# AF2811). After washing, membranes were incubated with their appropriate HRP-conjugated secondary antibodies (goat anti-mouse, Cat# A0350, Beyotime or goat anti-rabbit, Cat# A0208, Beyotime) for 1 h at room temperature. Protein bands were visualized using Efficient Chemiluminescence kit (Genview, Shanghai, China) and imaged with a ChemiDoc Touch Imaging System (Bio-Rad, Hercules, CA, USA). Band intensities were quantified in ImageJ and normalized to the corresponding β-actin bands.

**H&E stain**

Primary tissues (liver, spleen, and lower limb bones) were fixed in 4% paraformaldehyde (PFA), embedded in paraffin (SAV LP), and sectioned into 5 μm-thick slices. Hematoxylin and eosin (H&E) staining was performed using an H&E kit (Servicebio) following the manufacturer’s protocol. AML cells were identified based on characteristic morphological features, including enlarged and irregular cell shape, nuclear atypia and reduced cytoplasmic volume. The images were acquired using CaseViewer software (v.2.4).

**Immunofluorescence staining**

The dissected lower limb bones were fixed in 4% paraformaldehyde (PFA) and decalcified in 10% ethylenediaminetetraacetic acid for 2 weeks. Decalcified tissue was paraffin-embedded and made into 5 μm-thick sections. Antigen retrieval was performed using citrate buffer (pH 6.0) for 20 min. Endogenous peroxidase activity was quenched with 3% hydrogen peroxide (25 min, room temperature, light-protected), followed by blocking with 3% bovine serum albumin for 1 h. The tissue sections were then incubated in a humidified chamber at 4°C overnight with the following primary antibodies: anti-LEPR (Servicebio, Cat# GB112091), anti-Phospho-STAT3 (Ser705) (Servicebio, Cat# GB150001), anti-Phospho-JAK2 (Tyr1007/Tyr1008) (Servicebio, Cat# GB114585), anti-Phospho-STAT3 (Ser727) (Immunoway, Cat# YP0250). After washing, corresponding HRP-conjugated secondary antibodies (Servicebio, Cat# G1236-100T) were incubated for 50 min at room temperature. Signal amplification was achieved through tyramide signal amplification (TSA) reactions. Nuclear counterstaining with DAPI was performed followed by autofluorescence quenching. Whole-slide imaging was performed on a Pannoramic MIDI scanner (3DHISTECH), with multispectral unmixing of fluorophores using CaseViewer software (v2.4).

Cell lines were washed with PBS and uniformly seeded on glass coverslips, followed by fixation with 4% PFA for 15 min. After three PBS washes, samples were permeabilized with 0.2% Triton X-100 in PBS for 15 min and blocked with 2.5% goat serum for 20 min. Subsequently, cells were incubated overnight at 4°C with primary antibodies against LEPR (Proteintech, Cat# 20966-1-AP) or TOMM20 (Cell Signaling Technology, Cat# 42406S), respectively. After washing, they were incubated for 1 h at room temperature with the appropriate Alexa Fluor 488- (Beyotime, Cat# A0428) or Alexa Fluor 555-conjugated (Abcam, Cat# ab150078) secondary antibodies under light-protected conditions. Nuclei were counterstained with DAPI (5 μg/mL) for 3 minutes. After mounting with anti-fade medium, high-resolution images were acquired using a Leica TCS SP8 confocal microscope and analyzed using Leica LAS X software (v10.7.2). MFI of the indicated fluorescence signals was quantified by ImageJ software.

**Mitochondrial complex activity assays**

Following experimental interventions, approximately 1×10^6^ AML cells were washed with ice-cold PBS and resuspended in 100 μL assay buffer (provided in respective kits) supplemented with protease inhibitors. Cell suspensions were sonicated on ice (3 pulses of 10 seconds at 20% amplitude, with 30-second cooling intervals) to ensure complete cellular disruption while preserving enzyme activity. Lysates were clarified by centrifugation and proceed immediately to the enzymatic assays. The activities of mitochondrial respiratory chain complexes (I-V) were quantified by activity assay kits (all from Elabscience, Wuhan, Hubei, China) according to manufacturers' protocols.

**Total antioxidant capacity** **assays**

Following experimental treatments, approximately 1×10^6^ AML cells were resuspended in 200 μL of ice-cold PBS and lysed by ultrasonication. The lysates were centrifuged at 12,000 × g for 5 minutes at 4°C to remove cellular debris, and the resulting supernatants were immediately analyzed for total antioxidant capacity using the Total Antioxidant Capacity Assay Kit (Beyotime) according to the manufacturer's protocol.

**CAT and SOD activity assays**

Approximately 1×10^6^ AML cells were collected and resuspended in 200 μL of ice-cold assay buffer, followed by homogenization on ice using a homogenizer to ensure complete lysis of the cells while maintaining enzyme activity. Centrifuge the homogenate at 10,000 × g for 5 min at 4°C to remove cell debris and the resulting supernatants were immediately analyzed for CAT activity using the Catalase Activity Assay Kit (Abcam, Cambridge, UK) and SOD activity by the SOD Activity Assay Kit (Elabscience).

**GSH/GSSG ratio detection**

Approximately 1×10^6^ AML cells were resuspended in 30 μL assay buffer and subjected to two rapid freeze-thaw cycles (liquid nitrogen/37°C water bath) followed by 5-minute incubation on ice. After centrifugation, supernatants were collected for GSH/GSSG analysis using the GSH and GSSG Assay Kit (Beyotime) according to the manufacturer's protocol. Absorbance was measured at 412 nm using a microplate reader, with GSH/GSSG ratio calculated based on standard curves.

**Reverse transcription-quantitative PCR (RT-qPCR)**

Total RNA was isolated using TRIzol reagent and reverse transcribed using the 5×All-in-One RT MasterMix (Applied Biological Materials, Vancouver, Canada). RT-qPCR was performed using Eva Green 2×qPCR MasterMix-Low ROX (Applied Biological Materials). Gene expression levels were determined using the 2^-ΔΔCt^ method with Glyceraldehyde 3-phosphate dehydrogenase (*GAPDH*) serving as the endogenous reference control. RT-qPCR primer sequences are listed in Table S3.

**Leptin measurement in human and murine bone marrow**

For AML patients, bone marrow aspirates collected in heparinized tubes were centrifuged and plasma leptin levels were measured using a human leptin ELISA kit (Cusabio). For mice, bone marrow supernatant was obtained by flushing tibiae and femurs with 2 mL PBS followed by centrifugation, with leptin quantified using a Mouse Leptin ELISA Kit (Cusabio).

**Measurement of cellular oxygen consumption rate (OCR)**

OCR was measured using the Seahorse XF Cell Mito Stress Test Kit (Agilent Technologies, Santa Clara, CA) and the Seahorse XFe24 Analyzer (Agilent Technologies). For murine experiments, sorted YFP^+^ or GFP^+^ cells were seeded at 4 × 10^5^ cells per well in Cell-Tak-coated XFe24 microplates. AML cells lines were seeded at 2 × 10^5^ cells per well in identically coated plates. Cells were maintained in XF RPMI medium supplemented with 2 mM glutamine, 10 mM glucose, 1 mM pyruvate, and 5 mM HEPES in a CO_2_-free incubator for 30 min. The mitochondrial OCR was detected after sequential injection of 1 μM oligomycin, 2 μM carbonyl cyanide-4 (trifluoromethoxy) phenylhydrazone (FCCP) and 0.5 μM rotenone/antimycin A at specific time points.

**Quantitative proteomics of data-independent acquisition (DIA)**

YFP^+^ cells sorted from bone marrow of MLL-AF9 AML mice underwent protein extraction, reduction/alkylation, and tryptic digestion prior to ultra-high-performance liquid chromatography-tandem mass spectrometry (UHPLC-MS/MS) analysis. Raw data were processed via DIA-NN (v1.8.1) in library-free mode using the UniProtKB Mus musculus database (UP000000589; 54,910 sequences) to generate in silico spectral libraries, with deep neural networks predicting peptide fragmentation patterns and retention times. Match-between-runs (MBR) was enabled to enhance reproducibility, with precursor and protein-level false discovery rate (FDR) controlled at <1%. Differentially expressed proteins were identified by label-free quantification (fold-change > 1.2 or < 0.83, *p* < 0.05).

**Public database analysis**

Online Kaplan-Meier Plotter analysis (https://kmplot.com/analysis/) was employed to assess survival outcomes stratified by LEPR expression (high vs low) in AML datasets GSE1159, GSE37642, and GSE6891. Probe sets corresponding to the same gene were averaged, and patient cohorts were stratified using the automated optimal cutoff selection method.
