# Supplementary material for: Marrow leptin-LEPR signaling rewires mitochondrial oxidative metabolism to confer chemoresistance in acute myeloid leukemia
Source: Cell Death Dis. 2026 Feb 23;17(1):249. doi: 10.1038/s41419-026-08528-0 (PMC12966444; doi:10.1038/s41419-026-08528-0)
Supplement: Supplementary file 2 — Supplementary material [file 41419_2026_8528_MOESM2_ESM.pdf]

Western Blot Original Images

Figure 4A

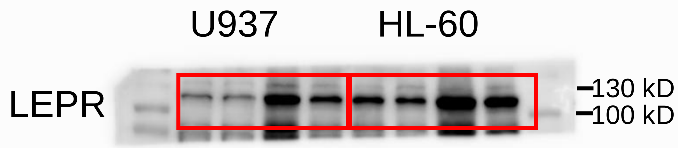
U937
HL-60
LEPR
130 kD
100 kD

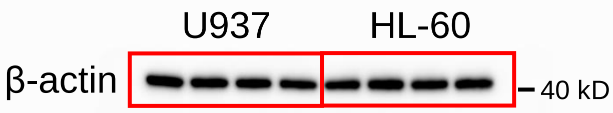
U937
HL-60
β-actin
40 kD

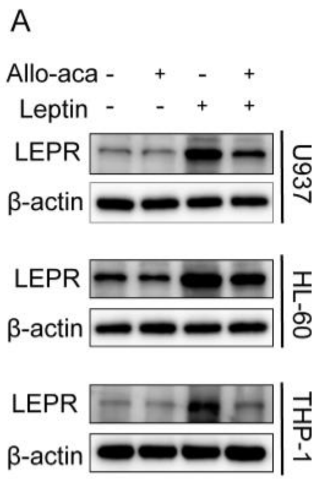
A
Allo-aca - + - +
Leptin - - + +
LEPR
β-actin
U937
LEPR
β-actin
HL-60
LEPR
β-actin
THP-1

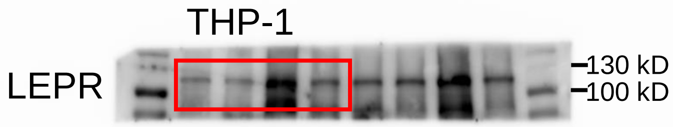
THP-1
LEPR
130 kD
100 kD

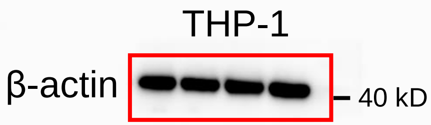
THP-1
β-actin
40 kD

Figure 4E

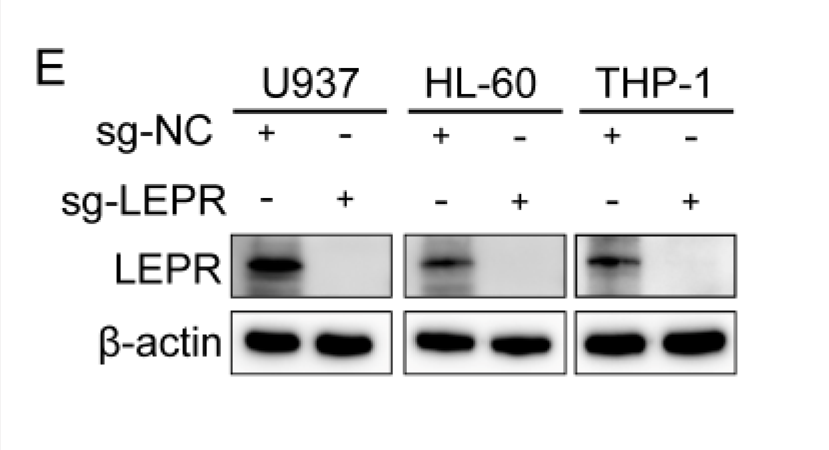
E
U937
HL-60
THP-1
sg-NC + - + - + -
sg-LEPR - + - + - +
LEPR
β-actin

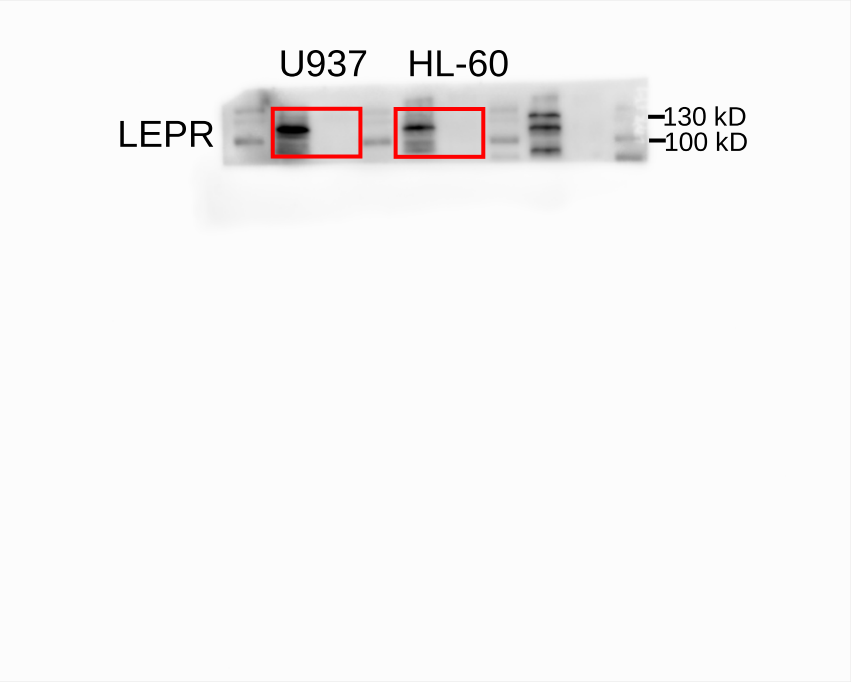
U937
HL-60
LEPR
130 kD
100 kD

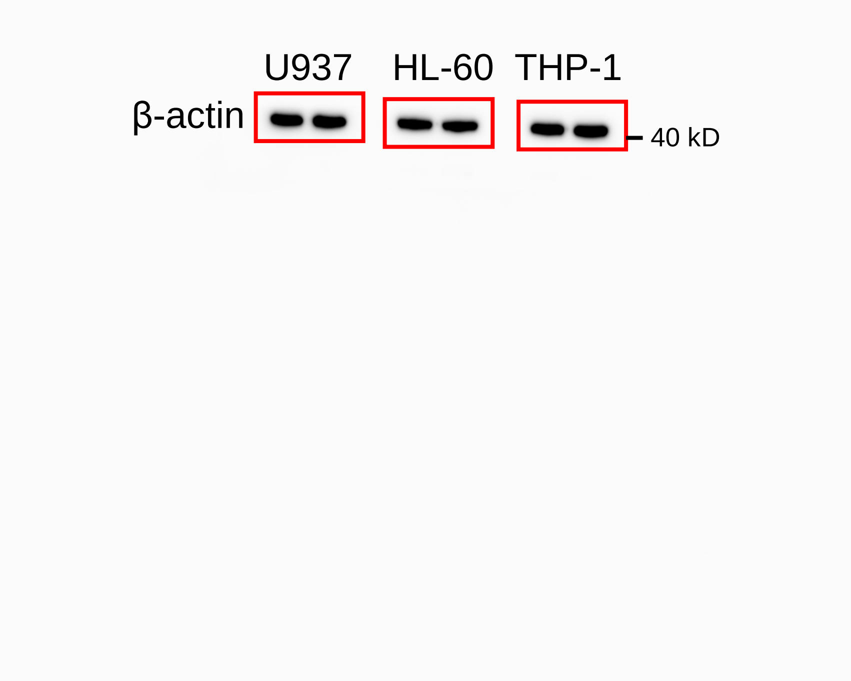
U937
HL-60
THP-1
β-actin
40 kD

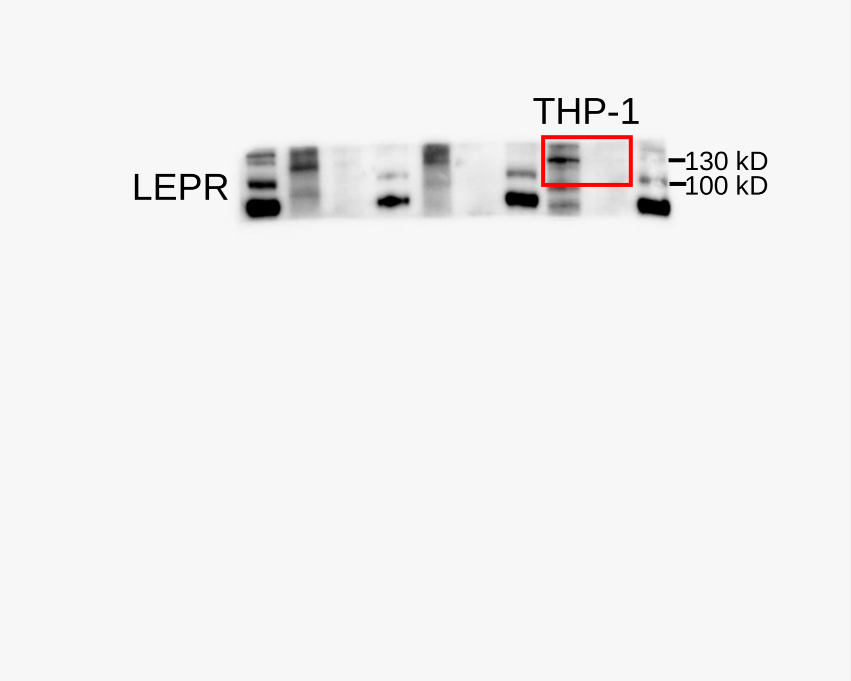
THP-1
LEPR
130 kD
100 kD

Figure 7E

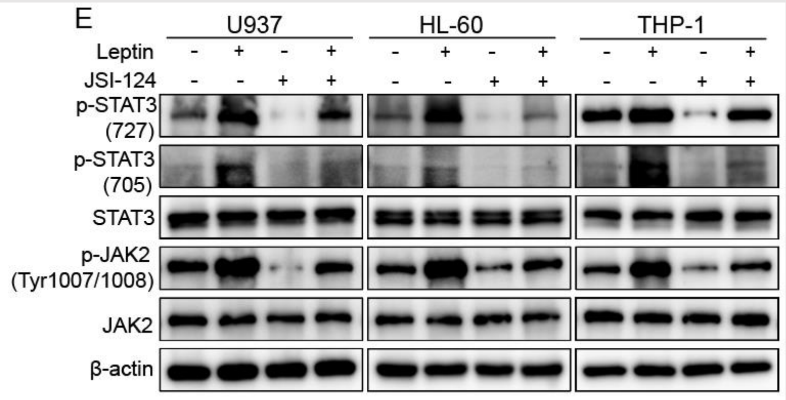
E
U937
HL-60
THP-1
Leptin - + - + - + - + - + - +
JSI-124 - - + + - - + + - - + +
p-STAT3 (727)
p-STAT3 (705)
STAT3
p-JAK2 (Tyr1007/1008)
JAK2
β-actin

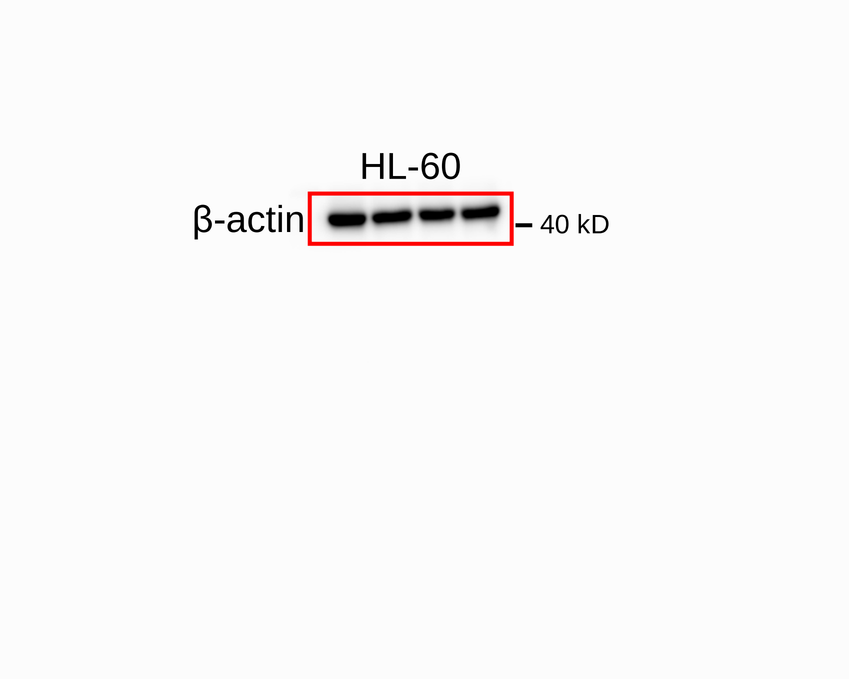
HL-60
β-actin
40 kD

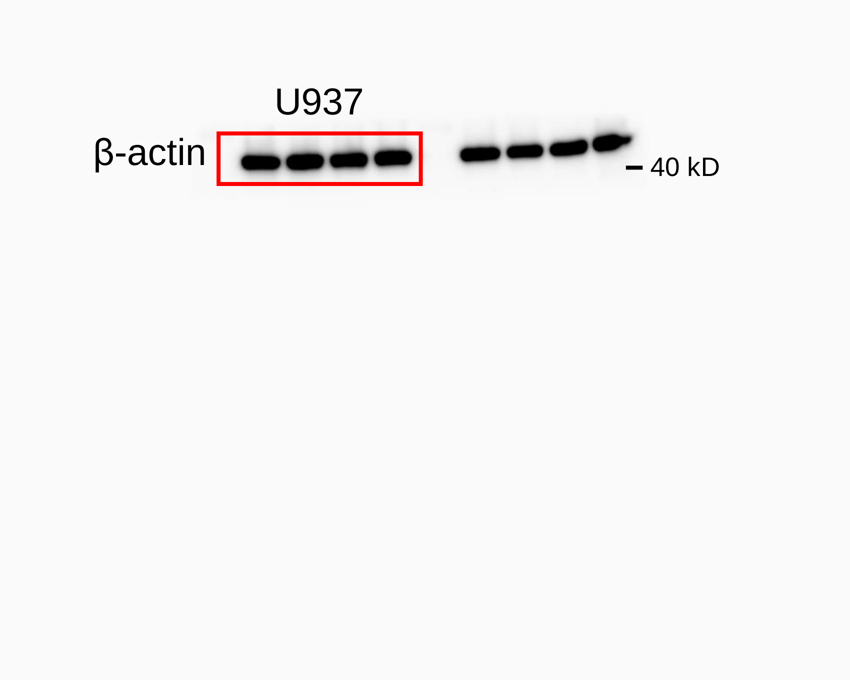
U937
β-actin
40 kD

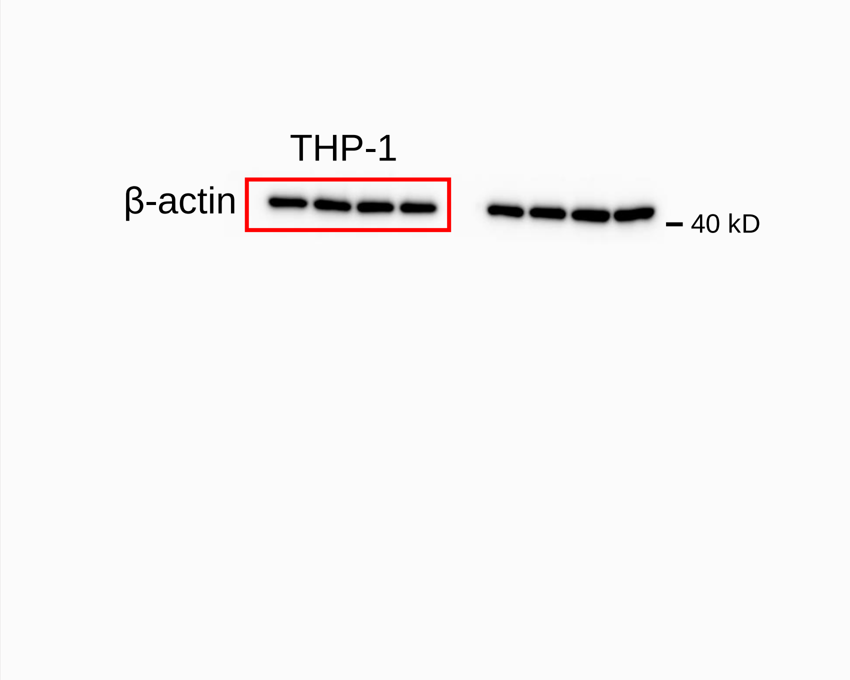
THP-1
β-actin
40 kD

Figure 7E

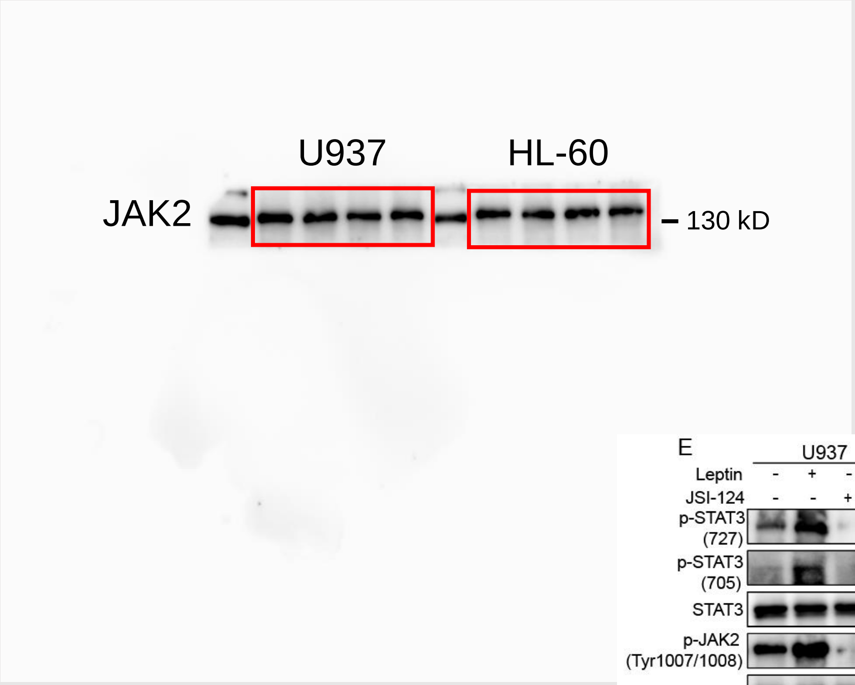
JAK2
U937
HL-60
130 kD

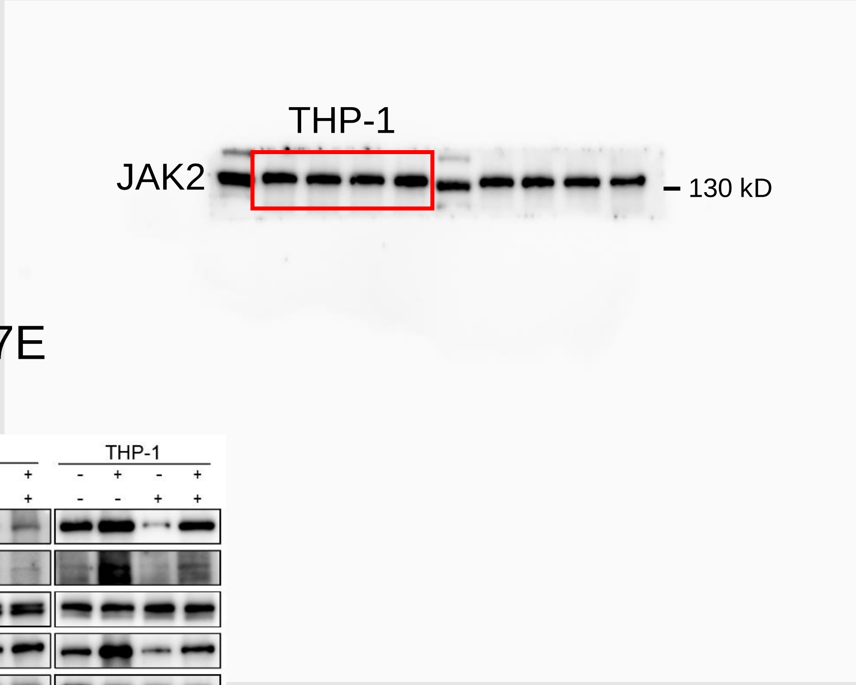
THP-1
JAK2
130 kD

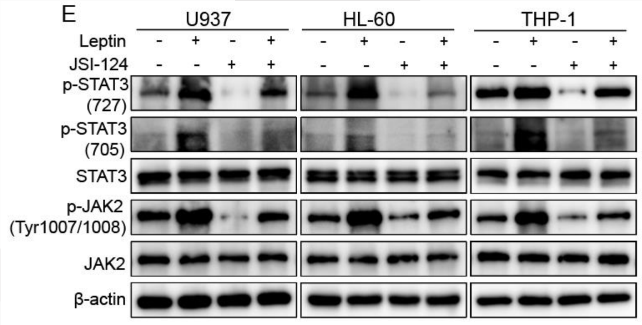
E
U937
HL-60
THP-1
Leptin
JSI-124
p-STAT3 (727)
p-STAT3 (705)
STAT3
p-JAK2 (Tyr1007/1008)
JAK2
β-actin

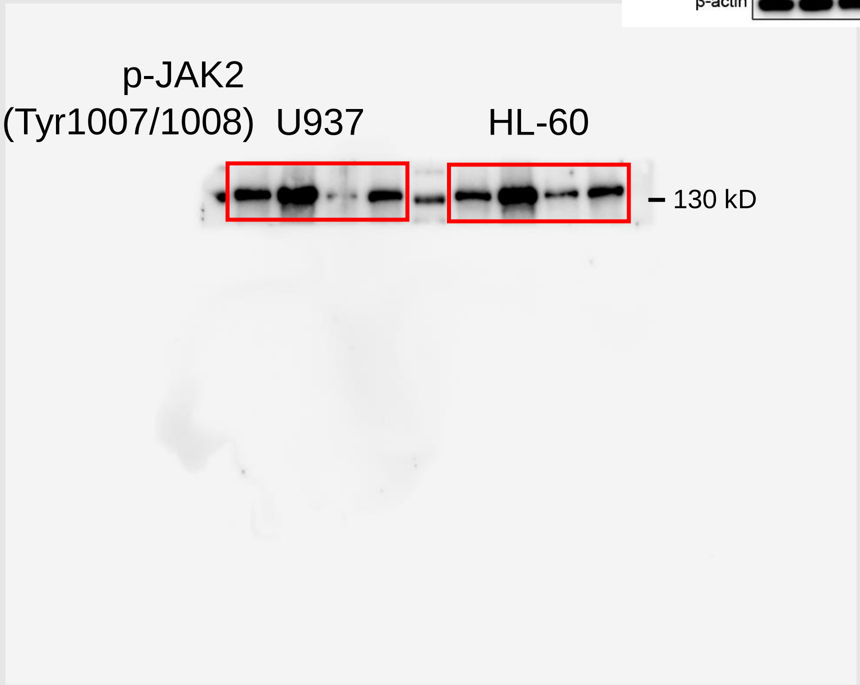
p-JAK2 (Tyr1007/1008)
U937
HL-60
130 kD

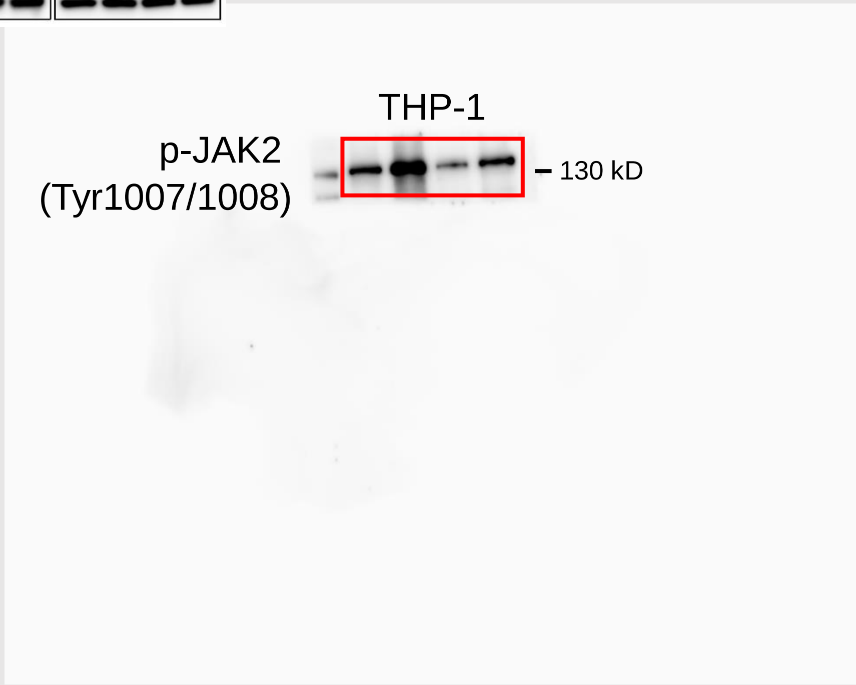
THP-1
p-JAK2 (Tyr1007/1008)
130 kD

Figure 7E

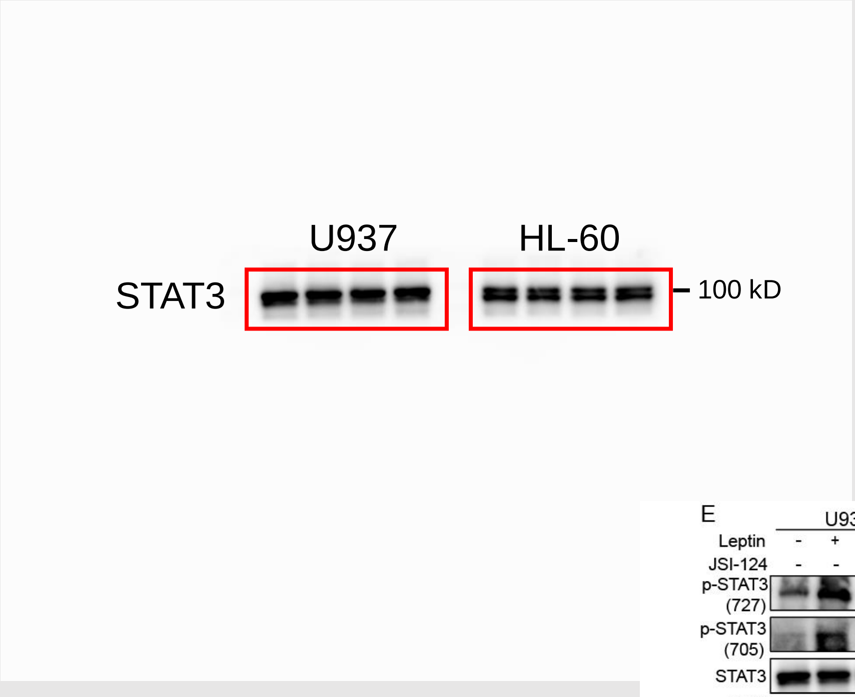
U937
HL-60
STAT3
100 kD

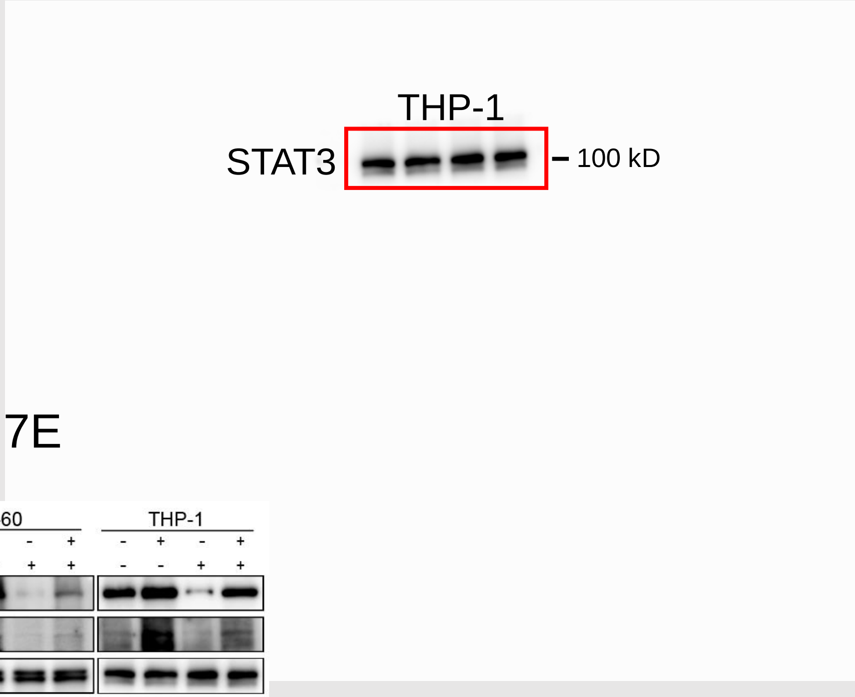
THP-1
STAT3
100 kD

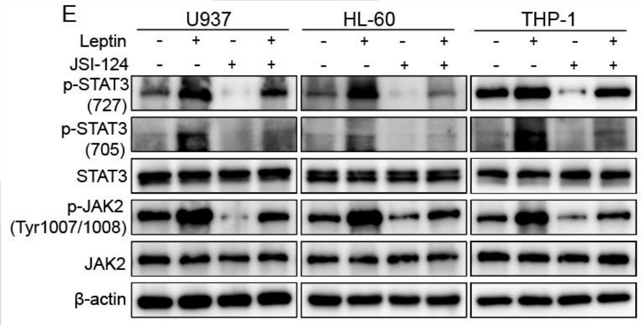
E
U937
HL-60
THP-1
Leptin - + - + - + - + - + - +
JSI-124 - - + + - - + + - - + +
p-STAT3 (727)
p-STAT3 (705)
STAT3
p-JAK2 (Tyr1007/1008)
JAK2
β-actin

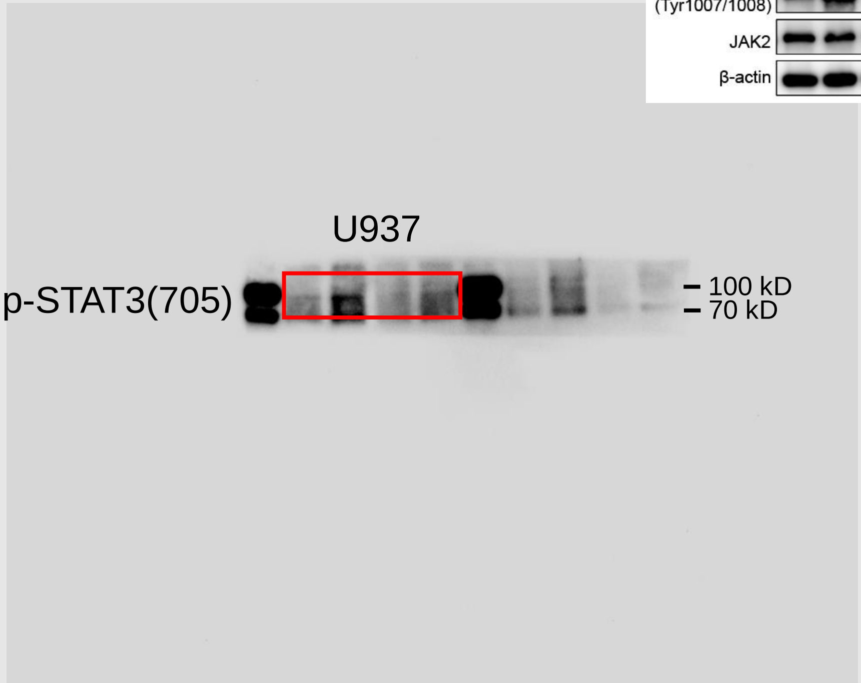
U937
p-STAT3(705)
100 kD
70 kD

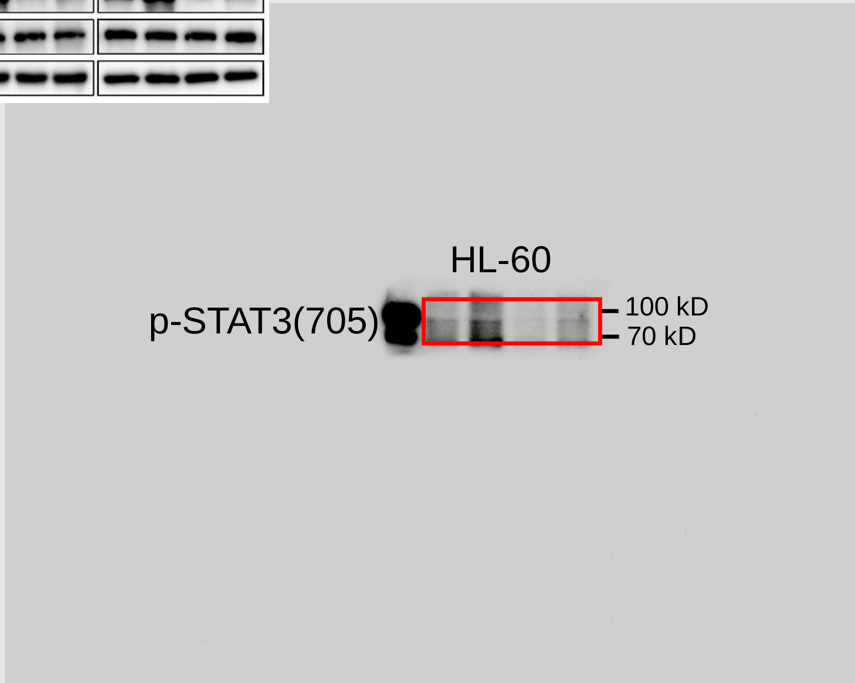
HL-60
p-STAT3(705)
100 kD
70 kD

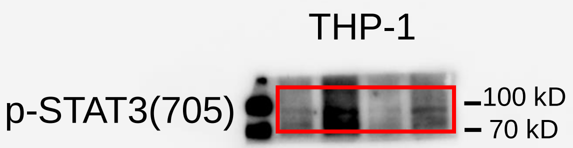
THP-1
p-STAT3(705)
100 kD
70 kD

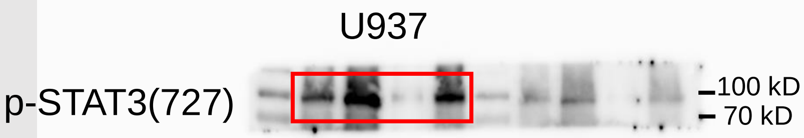
U937
p-STAT3(727)
100 kD
70 kD

Figure 7E

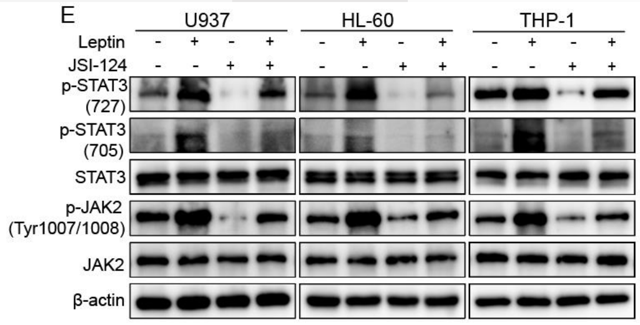
E
U937
HL-60
THP-1
Leptin - + - + - + - + - + - +
JSI-124 - - + + - - + + - - + +
p-STAT3 (727)
p-STAT3 (705)
STAT3
p-JAK2 (Tyr1007/1008)
JAK2
β-actin

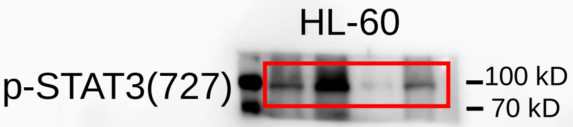
HL-60
p-STAT3(727)
100 kD
70 kD

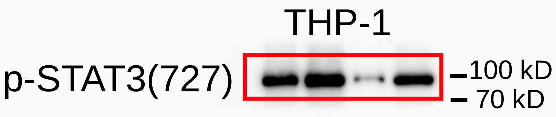
THP-1
p-STAT3(727)
100 kD
70 kD

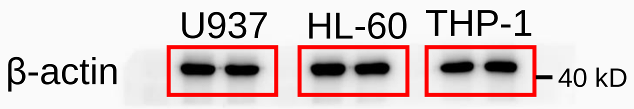
U937
HL-60
THP-1
β-actin
40 kD

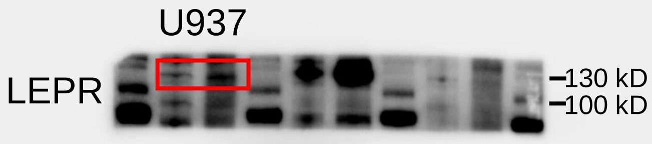
U937
LEPR
130 kD
100 kD

Figure S7G

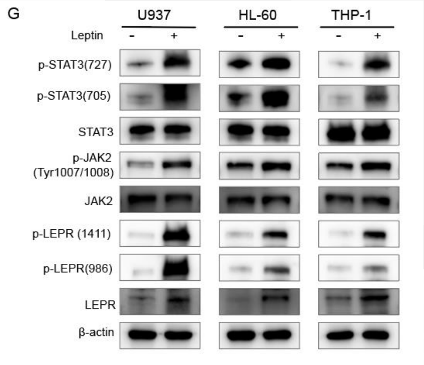
G
U937
HL-60
THP-1
Leptin
-
+
-
+
-
+
p-STAT3(727)
p-STAT3(705)
STAT3
p-JAK2
(Tyr1007/1008)
JAK2
p-LEPR (1411)
p-LEPR(986)
LEPR
β-actin

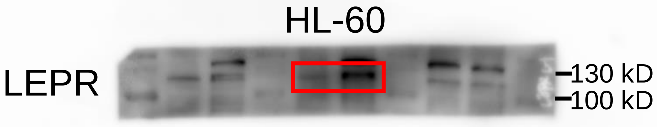
HL-60
LEPR
130 kD
100 kD

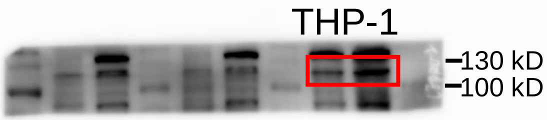
THP-1
130 kD
100 kD

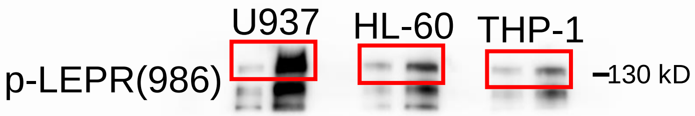
U937
HL-60
THP-1
p-LEPR(986)
130 kD

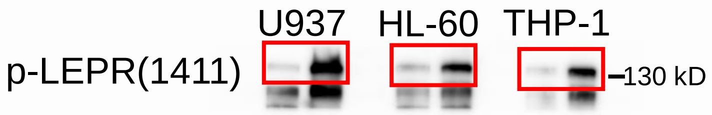
U937
HL-60
THP-1
p-LEPR(1411)
130 kD

Figure S7G

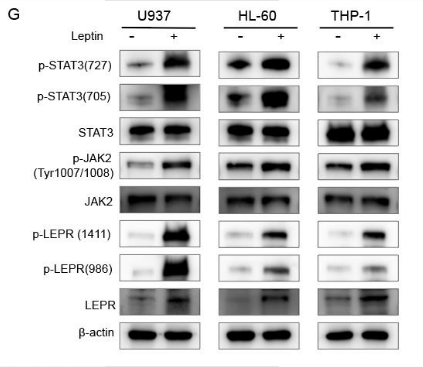
G
U937
HL-60
THP-1
Leptin
- +
- +
- +
p-STAT3(727)
p-STAT3(705)
STAT3
p-JAK2
(Tyr1007/1008)
JAK2
p-LEPR (1411)
p-LEPR(986)
LEPR
β-actin

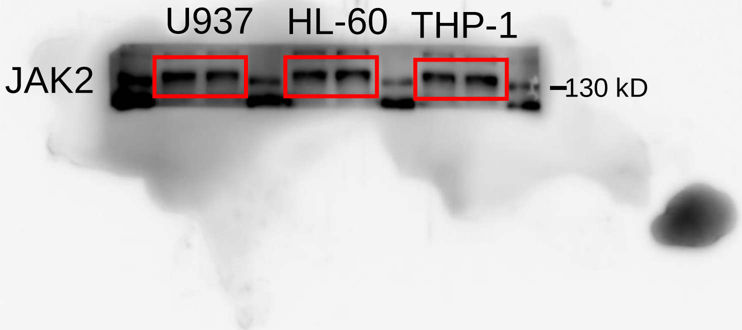
U937
HL-60
THP-1
JAK2
130 kD

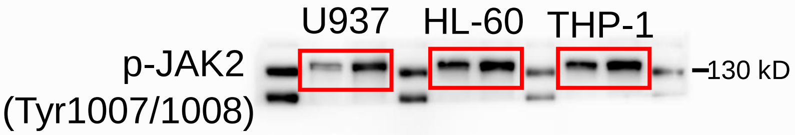
U937
HL-60
THP-1
p-JAK2
(Tyr1007/1008)
130 kD

Figure S7G

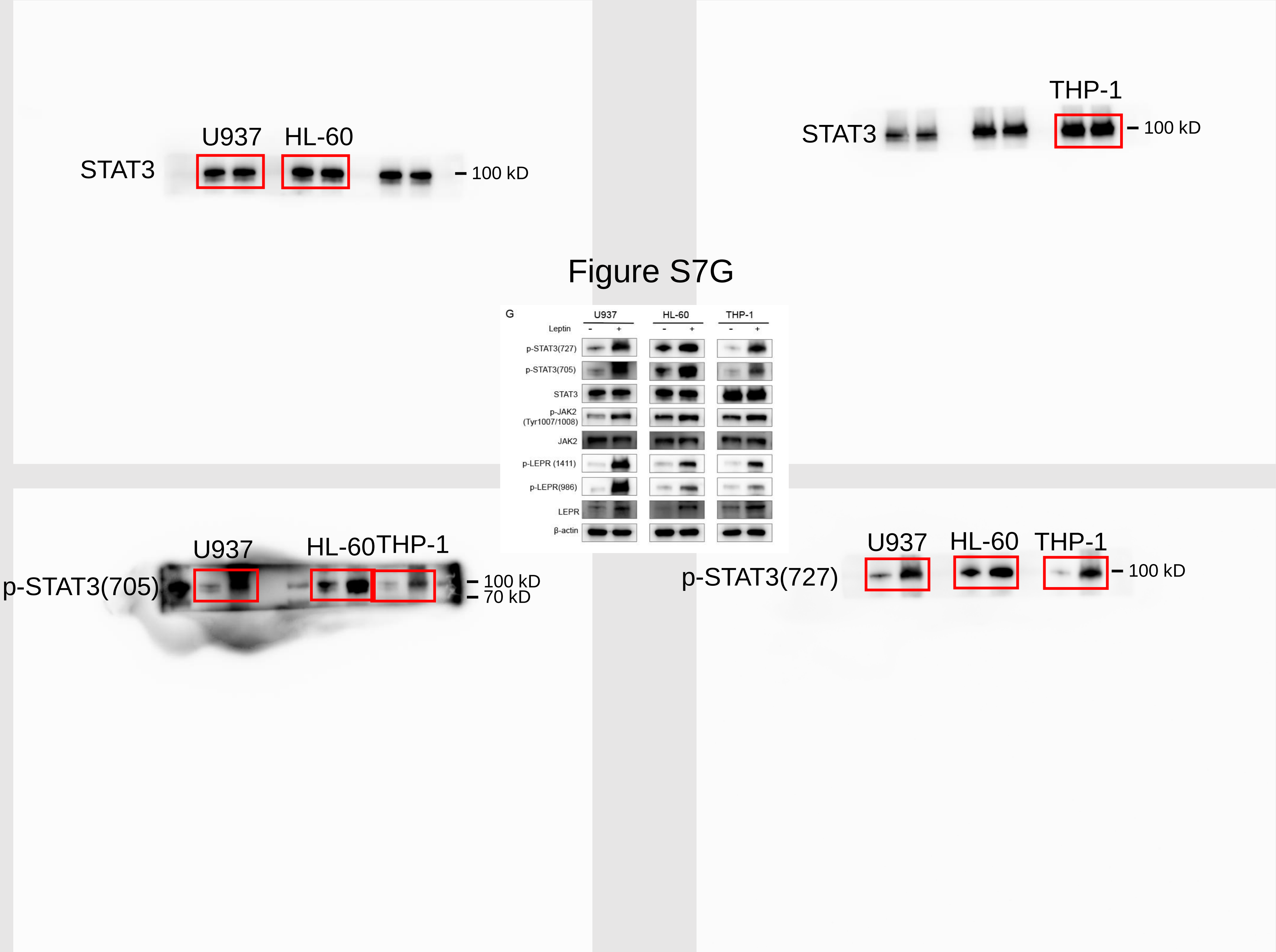

STAT3
U937
HL-60
100 kD
STAT3
THP-1
100 kD
G
U937
HL-60
THP-1
Leptin
- + - + - +
p-STAT3(727)
p-STAT3(705)
STAT3
p-JAK2 (Tyr1007/1008)
JAK2
p-LEPR (1411)
p-LEPR(986)
LEPR
β-actin
p-STAT3(705)
U937
HL-60
THP-1
100 kD
70 kD
p-STAT3(727)
U937
HL-60
THP-1
100 kD

Figure S7H

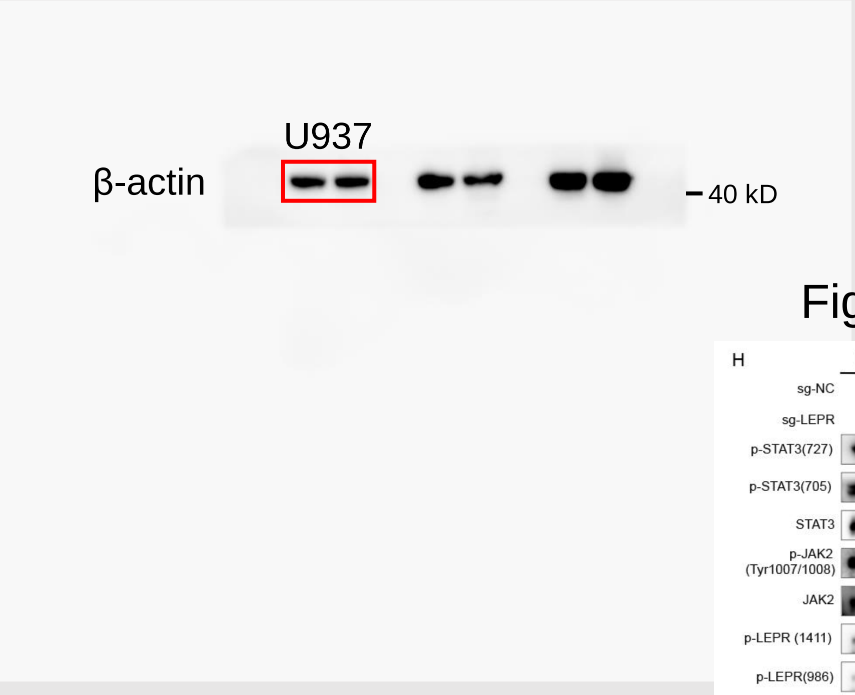
U937
β-actin
40 kD

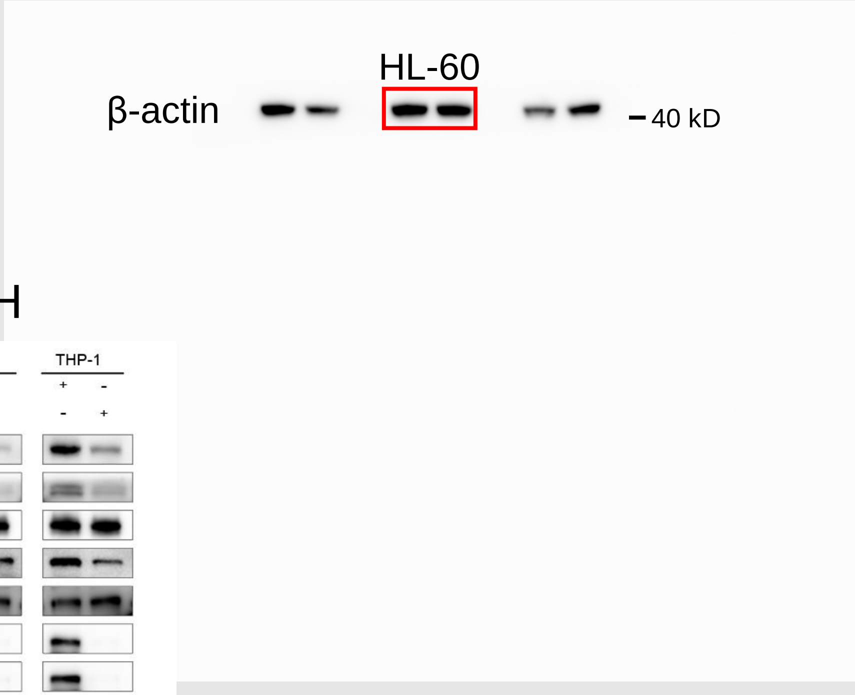
HL-60
β-actin
40 kD

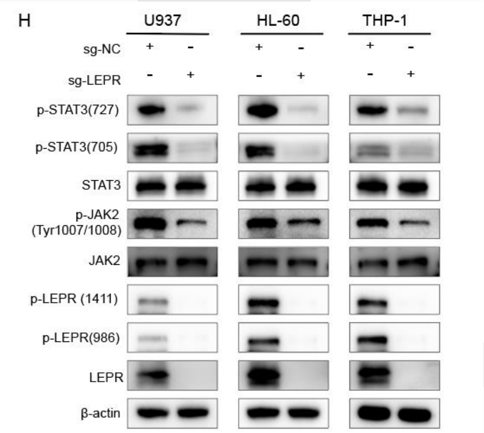
H
U937
HL-60
THP-1
sg-NC
sg-LEPR
p-STAT3(727)
p-STAT3(705)
STAT3
p-JAK2 (Tyr1007/1008)
JAK2
p-LEPR (1411)
p-LEPR(986)
LEPR
β-actin

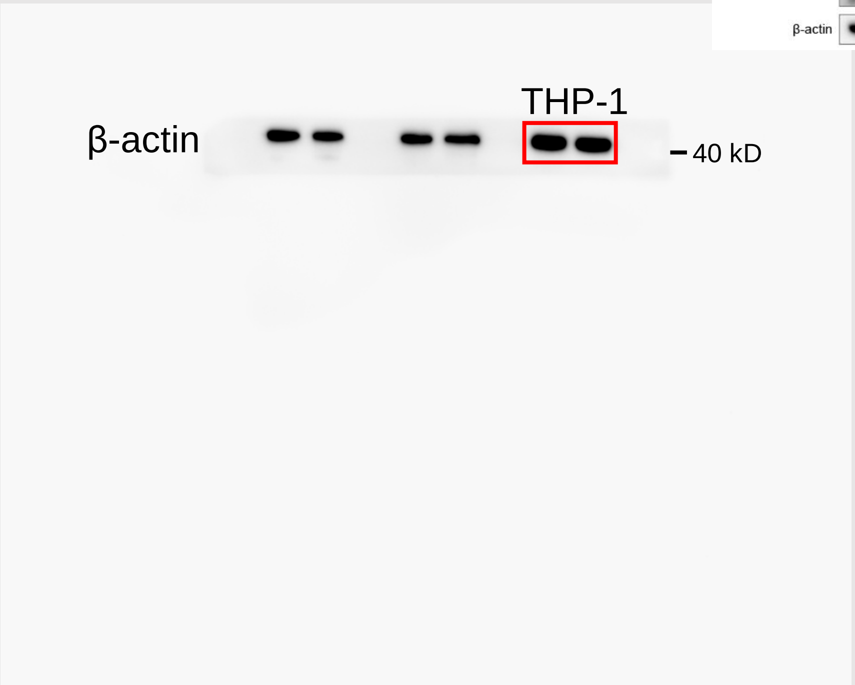
THP-1
β-actin
40 kD

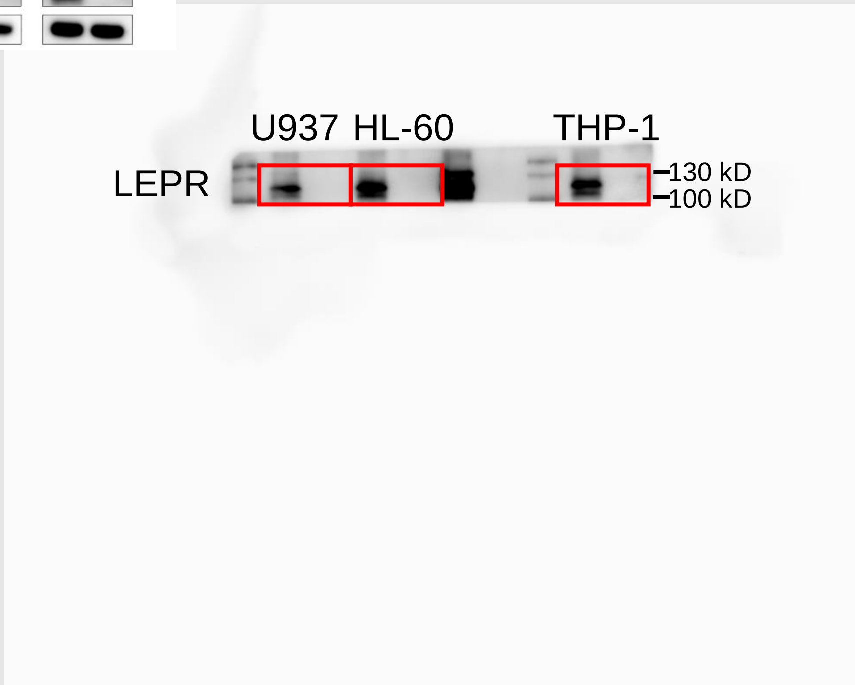
U937
HL-60
THP-1
LEPR
130 kD
100 kD

Figure S7H

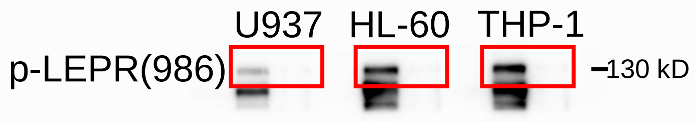

p-LEPR(986)
U937
HL-60
THP-1
130 kD

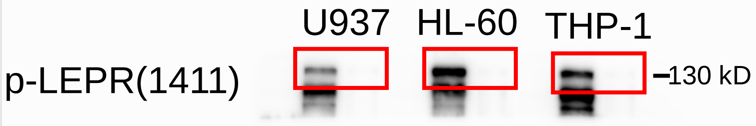

p-LEPR(1411)
U937
HL-60
THP-1
130 kD

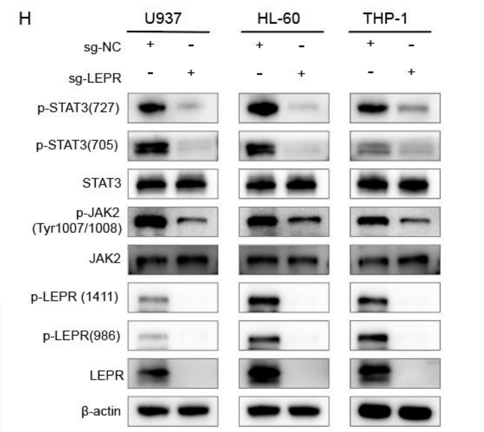

H
U937
HL-60
THP-1
sg-NC
sg-LEPR
p-STAT3(727)
p-STAT3(705)
STAT3
p-JAK2 (Tyr1007/1008)
JAK2
p-LEPR (1411)
p-LEPR(986)
LEPR
β-actin

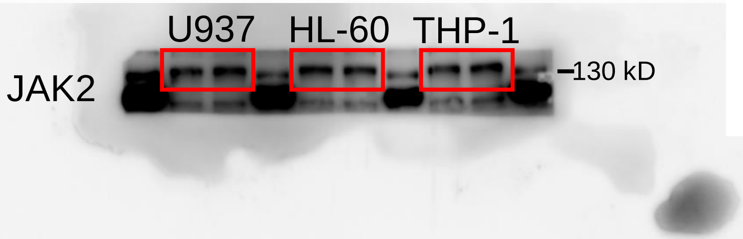

JAK2
U937
HL-60
THP-1
130 kD

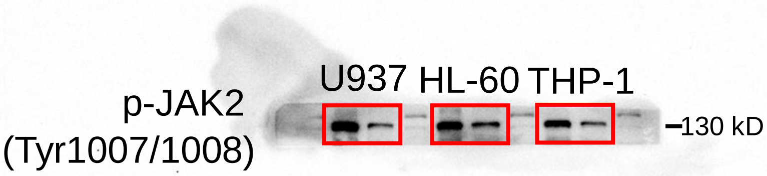

p-JAK2 (Tyr1007/1008)
U937
HL-60
THP-1
130 kD

Figure S7H

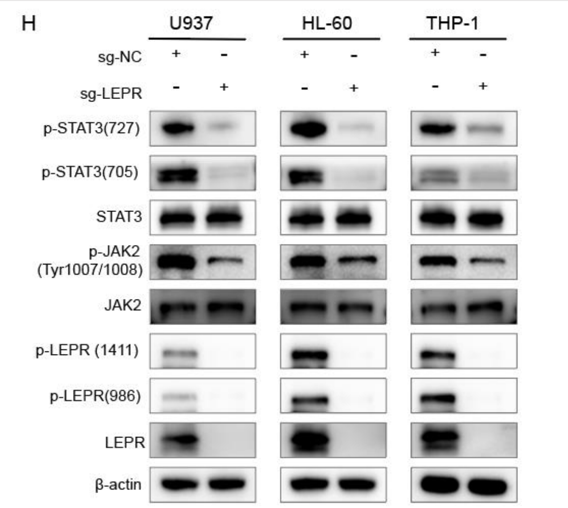
H
U937
HL-60
THP-1
sg-NC + - + - + -
sg-LEPR - + - + - +
p-STAT3(727)
p-STAT3(705)
STAT3
p-JAK2 (Tyr1007/1008)
JAK2
p-LEPR (1411)
p-LEPR(986)
LEPR
β-actin

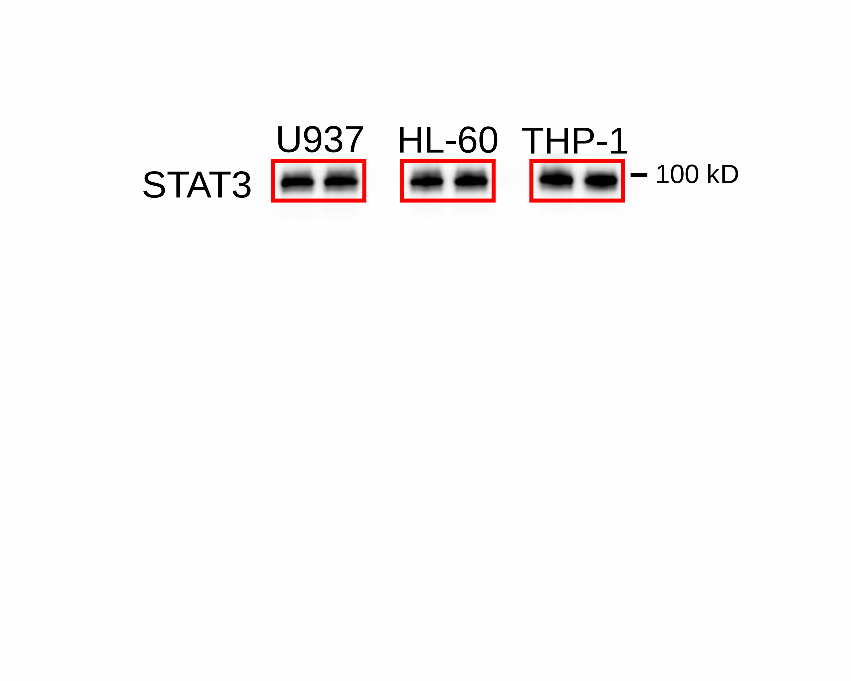
U937
HL-60
THP-1
STAT3
100 kD

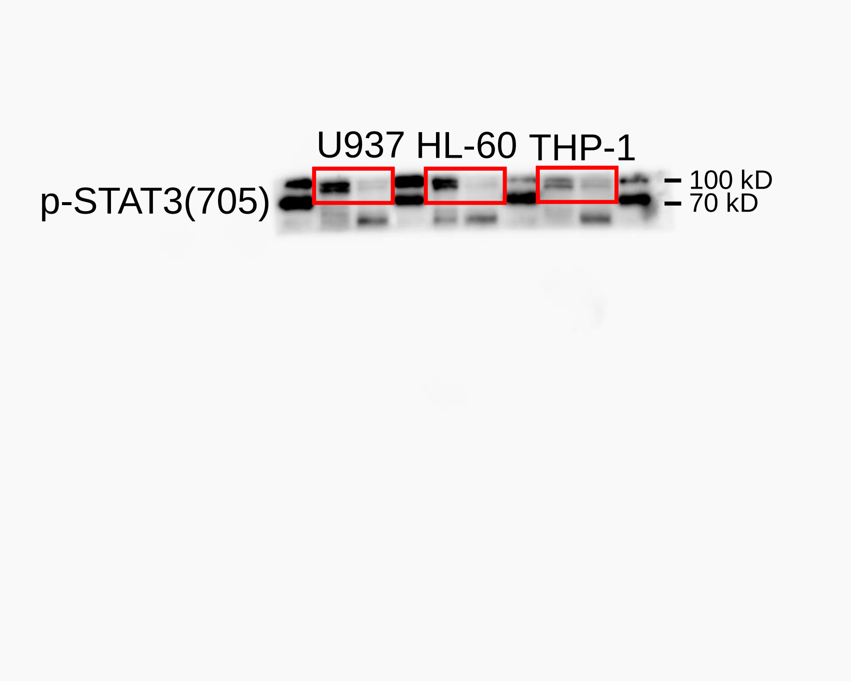
U937 HL-60 THP-1
p-STAT3(705)
100 kD
70 kD

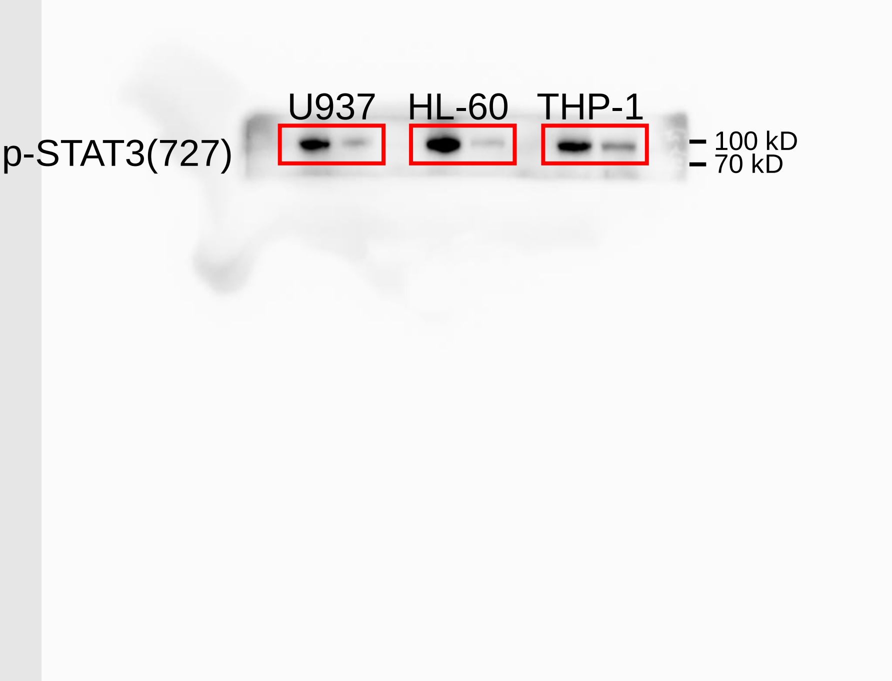
U937
HL-60
THP-1
p-STAT3(727)
100 kD
70 kD
